# A meta-analysis on the efficacy of low-intensity cognitive behavioural therapy for generalised anxiety disorder

**DOI:** 10.1186/s12888-023-05306-6

**Published:** 2024-01-02

**Authors:** Candice L. Y. M. Powell, Chun Yuen Chiu, Xiaoqi Sun, Suzanne Ho-wai So

**Affiliations:** 1 Clinical Psychological Services, New Life Psychiatric Rehabilitation Association, Hong Kong Special Administrative Region, China; 2 Department of Psychology, Hunan Normal University, Hunan, China; 3 Cognition and Human Behavior Key Laboratory of Hunan Province, Hunan Normal University, Hunan, China; 4 Department of Psychology, The Chinese University of Hong Kong, Room 321, Wong Foo Yuan Building, Shatin, Hong Kong SAR, China

**Keywords:** Generalised anxiety, Worry, Depression, Stepped care, Self-help, Psychoeducation, Review, GAD, NICE guideline

## Abstract

**Background:**

Low-intensity cognitive behavioural therapy (LICBT) has been recommended as a primary intervention in the tiered care for mild to moderate generalised anxiety disorder. However, LICBT for generalised anxiety disorder are markedly diverse and efficacy data on various outcomes have not been systematically reviewed. This meta-analysis aimed to synthesise effect sizes of three NICE-recommended LICBT for generalised anxiety disorder: non-facilitated self-help, guided self-help, and psychoeducational groups.

**Methods:**

A systematic literature review of randomised controlled trials (RCTs) examining LICBT for generalised anxiety disorder in the last 23 years (2000–2023) was conducted. Efficacy data for anxiety, depression, and worry outcomes were separately meta-analysed. The study was reported following the PRISMA guidelines.

**Results:**

The systematic review identified 12 RCTs out of 1205 papers. The three meta-analyses consisted of 12 (anxiety), 11 (depression), and 9 (worry) effect sizes respectively, including total sample sizes of 1201 (anxiety), 1164 (depression), and 908 (worry). The adjusted effect sizes for reductions in anxiety (*g* = -0.63), depression (*g* = -0.48), and worry (*g* = -0.64) were all in the medium range, favouring LICBT over control conditions. Between-study heterogeneity was significant on anxiety and worry, with no specific moderators identified by meta-regression.

**Conclusions:**

LICBT has shown promise as an effective and efficient treatment modality for individuals with generalised anxiety disorder. Future research comparing various LICBT subtypes and treatment components will further inform clinical practice.

**Trial registration:**

This systematic review protocol has been registered with the International Prospective Register of Systematic Reviews (PROSPERO; record ID CRD42021285590).

**Supplementary Information:**

The online version contains supplementary material available at 10.1186/s12888-023-05306-6.

## Background

Generalised anxiety disorder (GAD) is characterized by excessive and uncontrollable anxiety and worry about everyday internal and external events. Its lifetime and 12-month prevalence rates are 3.7% and 1.8% respectively, with rates generally higher in higher-income countries [1]. In addition to anxiety, worry, and their associated distressing physical symptoms, more than 80% of individuals with GAD have a comorbid mental disorder, with major depressive disorder being the most common comorbidity [1–3]. It is typical for patients to still be affected 6–12 years after onset, with only 14–39% of them attaining full recovery [4–7]. GAD predicts a 77% increase in premature mortality and cardiovascular deaths [8, 9], and more than half of individuals with GAD are severely disabled [1, 10, 11].

In view of the prevalence, chronicity, and impairment of GAD, it is of public health interest to develop efficacious and cost-effective interventions [12–14]. In accordance with the stepped care model for common mental disorders, services have recently been developed in a way that service users are triaged into low- and high-intensity interventions according to their clinical presentations [15]. Unlike high-intensity interventions, low-intensity interventions consist of fewer therapeutic components, are shorter in duration, and can be delivered by non-specialist practitioners who are specifically trained and supervised. The stepped care model ensures that the right kind of intervention is accessed by targeted service users efficiently, curtailing the waiting list. Service users with milder symptoms may receive low-intensity interventions only, whereas those with more severe symptoms will be stepped up to high-intensity services provided by specialist therapists, allowing flexibility [16]. The stepped care approach for anxiety disorders (including GAD) has been adopted and tested in various mental health systems, such as the UK, Australia and Hong Kong [17–19].

According to the NICE [20] guidelines, education and active monitoring is recommended as Step 1 intervention for GAD in the UK. Individuals who have not improved after Step 1 should receive low-intensity (LI) interventions at Step 2, and either high-intensity (HI) psychological intervention or drug treatment at Step 3 if needed [15]. In particular, high-intensity cognitive behavioural therapy (HICBT) is recommended as a form of Step 3 intervention, whereas Step 2 interventions should make use of written or electronic materials based on CBT treatment principles, which may be delivered in a guided or non-facilitated manner.

While HICBT for GAD is well established and tested, LICBT strategies for GAD are less so. HICBT for GAD typically consists of 12–18 sessions with therapeutic components such as psychoeducation, relaxation training, exposure and behavioural experiments, and cognitive restructuring. Traditional CBT considered as ‘high intensity’ targets cognitive and behavioural processes that maintain the worry process and anxiety symptoms, so that individuals revisit their beliefs about worry, reduce avoidance behaviour and improve coping [21–24]. Moderate-to-large effect sizes have been reported for HICBT in reducing anxiety, worry, and depression compared with a control condition [25–28].

On the contrary, LICBT for GAD has only been recently developed, with marked diversity in therapeutic components, delivery modes, and treatment duration. The NICE guidelines recommend the following three types of Step 2 CBT-based interventions for GAD: (i) individual non-facilitated self-help, (ii) individual guided self-help, and (iii) psychoeducational groups, which can be delivered face-to-face, in a video conference, or over the phone. The duration of the intervention can range from five to seven weeks, with contact time of five minutes to two hours per week provided by supervised practitioners [20]. On the other hand, in Australia, LI interventions in general consist of no more than eight sessions [29]. Shafran, Myles-Hooton, Bennett, and Öst [30] defined LI interventions as the utilization of self-help materials with six or fewer contact hours in total, provided by trained practitioners or supporters in various formats. None of these guidelines or definitions specifies the CBT-based therapeutic components.

Without an agreed definition of LICBT for GAD, reviews on this topic have been sparse, with comparisons made across a small number of studies on diverse and anecdotal parameters only. For example, reviews that focused on the mode of delivery showed that Internet-based treatment had equally large effect sizes on anxiety, worry, and depression [31] as face-to-face therapies [26, 32, 33]. Haug and colleagues [34] reported that guided self-help tends to be superior to pure self-help for all kinds of anxiety disorders. Focusing on the duration of treatment, Hunot and colleagues [35] reported that reduction in anxiety symptoms was comparable between shorter (< 8 sessions) and longer (≥ 8 sessions) regimens, whereas reduction in worry and depression was only effective following longer regimens.

Therefore, the current meta-analysis aimed to provide an up-to-date quantitative integration of efficacies of LICBT for GAD, taking into account the variety of service delivery within a clear and coherent framework, so that treatment trial data can be synthesised and compared in a meaningful way. Separate meta-analyses were conducted for each of the three outcome constructs: anxiety, worry, and depression. We hypothesised that (i) all three types of LICBT (as specified by the NICE guidelines) will be superior to control conditions in reducing anxiety, worry, and depression, and that (ii) guided self-help and psychoeducational groups will be more efficacious than non-facilitated self-help.

## Methods

This study followed the Preferred Reporting Items for Systematic Reviews and Meta-Analyses (PRISMA) guidelines [36]. This systematic review protocol has been registered with the International Prospective Register of Systematic Reviews (PROSPERO; record ID CRD42021285590).

### Search strategy

A systematic search of the literature was performed on three electronic databases (PubMed, PsycINFO, and Cochrane library). The search was carried out in October 2021 and updated in July 2023. The search was limited to peer-reviewed articles published within the past 23 years (January 2000 to July 2023). In order to represent the current literature comprehensively, the current meta-analysis adopted an inclusive set of selection criteria: no more than eight sessions in total, with no more than two hours per session, and treatment components aligning with CBT principles (e.g. psychoeducation, use of worry time, guided problem solving, relaxation techniques, cognitive restructuring, exposure, and other behavioural strategies). Only randomised controlled trials with a GAD sample were included.

The search strategy included search terms that represented the following key concepts: (i) generalised anxiety disorder (“generalis[z]ed anxiety disorder” OR “GAD”); (ii) low-intensity (“low-intensity” OR “guided” OR “self-help” OR “computeris[z]ed” OR “internet” OR “mobile” OR “app”); (iii) CBT (“CBT” OR “iCBT” OR “cCBT” OR “cognitive” OR “behavio[u]r*”); and (iv) trial (“trial” OR “controlled” OR “RCT” OR “randomis[z]ed”). These four concepts were linked by the Boolean operator “AND”.

**Fig. 1:**
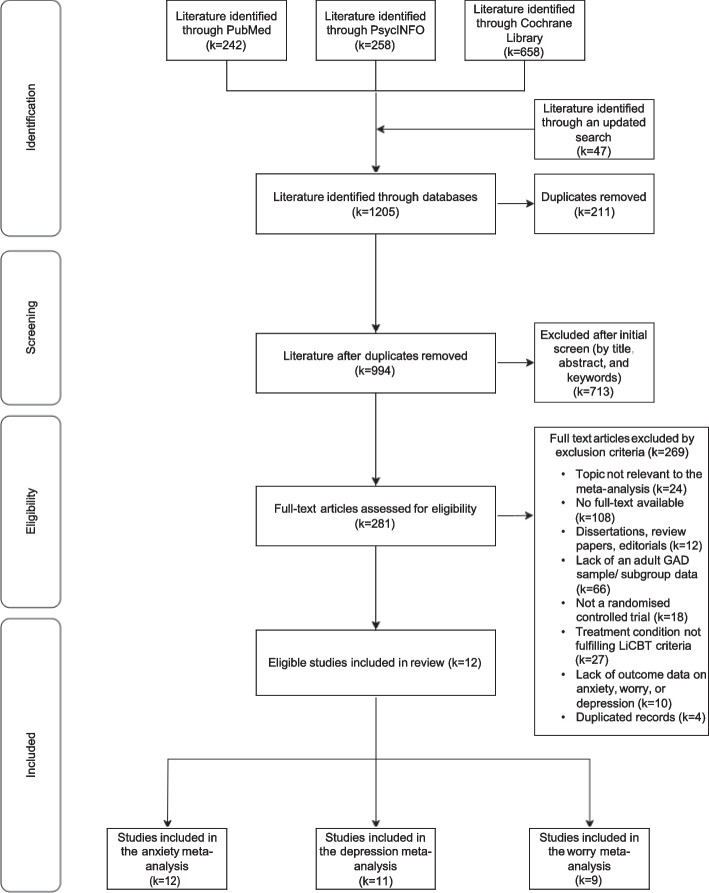
PRISMA flow diagram illustrating the literature search and selection procedure

After the primary search was completed, secondary searches were conducted by screening through reference lists of the included studies and two major journals in the area of interest (i.e., *Journal of Consulting and Clinical Psychology* and *Behaviour Research and Therapy*), with the same publication period applied. The procedure of study identification and selection is presented in Fig. 1.

### Inclusion and exclusion criteria

Inclusion criteria were as follows: (1) adult participants (18 years old or above), (2) participants with GAD according to The Diagnostic and Statistical Manual of Mental Disorders (DSM), International Classification of Diseases (ICD), or other validated clinical tools with established clinical thresholds, (3) an intervention that fulfilled the criteria of LICBT in terms of treatment duration and content, (4) availability of outcome data on anxiety, worry, or depression (or their combinations), (5) a randomised controlled trial (with a non-LICBT control condition), and (6) publication in English. Studies were excluded if no full-text was available, or if the work was neither empirical nor peer-reviewed (e.g. dissertations, review papers, editorials, etc.).

### Selection of studies

Study selection was performed by three research workers (XS, JH, and CYC) independently. Disagreements were discussed and resolved by consensus among the authors at every step of the process. After an initial screen based on study titles and abstracts, full texts of potentially eligible studies were inspected against the selection criteria. A secondary search was performed by JH and CYC. When the data necessary for calculating the effect sizes were not provided in the papers, the authors of the respective papers were contacted.

### Data extraction

Table 1 displays the following study characteristics: name, year, location, sample size and characteristics, targeted condition(s), diagnostic assessment, LICBT treatment modality, and outcome measures.

**Table 1:** Study characteristics (*k* = 12)

Study	Location	Sample	Target condition(s)	Eligibility based on	Content of LICBT	Type of LI CBT	Anxiety measure(s)	Depression measure(s)	Worry measure(s)
Al-Alawi et al. (2021) [37*]	Oman	n = 46; GAD-7 total score ≥ 12 or PHQ-9 total score ≥ 10	COVID-19–induced anxiety and depression	non-specified assessor	Therapist-Guided Online Therapy Versus Self-help Internet-Based Therapy; 6 weeks	individual	GAD-7	PHQ-9	
Andersson et al. (2012) [38*]	Sweden	n = 81; Structured Clinical Interview for DSM-IV Axis I Disorders research version – SCID-I	GAD	trained assessor	Internet-Based Cognitive Behavioral Therapy; 8 modules over 8 weeks; total therapist contact = 92 min	individual-guided	BAI, STAI-S, STAI-T, GAD-Q-IV	BDI-II	PSWQ
Carl et al. (2020) [39*]	USA, UK	n = 256; GAD 7 ≥ 10, combined with a GAD diagnosis with a digital MINI-7 for DSM-5	GAD	trained assessor	Daylight, smartphone-based and fully automated digital CBT intervention. 4 modules over 6 weeks (10–20 min/per session). The program was self-paced and delivered through a cellphone	individual-guided	GAD-7	PHQ-9	PSWQ
Dahlin et al. (2016) [40*]	Sweden	n = 103; PSWQ ≥ 45, MADRS-S ≤ 30, SCID-I	GAD	trained assessor	“Oroshjälpen” (translated “The anxiety aid”). 7 modules over 9 weeks; no information about session duration	individual	GAD-7	PHQ-9	PSWQ
Jones et al. (2016) [41*]	Canada	n = 46, GAD-7 ≥ 10, MINI and MINI Plus; 60 years old or above	clinical, subclinical GAD	trained assessor and clinician	GAD Online for Older Adults, ICBT programs for GAD. 7 modules over 10 weeks, no information about session duration	individual-guided	GAD-7, GAI	PHQ-9, GDS	PSWQ-A
Newman et al. (2014) [42*]	USA	n = 38, with a principal diagnosis of GAD using ADIS-IV	GAD	trained assessor	Palmtop computer-assisted Group CBT for GAD, or Group CBT for GAD without the computer. 6 sessions (120 min each) over 8 weeks	group	HAM-A, STAI-T		PSWQ
Paxling et al. (2011) [43*]	Sweden	n = 89, telephone interview with SCID-I	GAD	trained assessor, confirmed by clinician	Guided Internet-delivered cognitive behavior therapy. 8 modules over 8 weeks. Variable session duration	individual-guided	GAD-Q-IV, STAI, BAI	MADRS-S, BDI	PSWQ
Richards et al. (2016) [44*]	Ireland	n = 137, GAD-7 ≥ 10	GAD	self-report	The Calming Anxiety supported programme, an Internet-delivered CBT intervention. 6 sessions over 6 weeks; mean session duration 27.38 min	individual-guided	GAD-7	BDI-II	PSWQ
Robinson et al. (2010) [45*]	Australia	n = 150, telephone interview with MINI 5.0.0	GAD	non-specified assessor	The Worry program, an iCBT program. Technician-assisted or clinician-assisted. 6 online lessons within 10 weeks; no information on session duration	individual	GAD-7	PHQ-9	PSWQ
Rollman et al. (2017) [46*]	USA	n = 155, GAD-7 ≥ 10, 18–75 years old	Mood and anxiety disorders	self-report	Care manager-guided, the Beating the Blues CCBT program. 8 sessions over six months, 1 to 2 sessions per week; each session lasted for 60 min	individual-guided	PROMIS	PROMIS	
Terides et al. (2017) [47*]	Australia	n = 89; first screened (GAD-7 ≥ 5 or PHQ-9 ≥ 5), then MINI-5	Depression, anxiety	trained assessor	The Wellbeing Course, iCBT (a wellbeing course). 8 weeks; no information about session duration	individual-guided	GAD-7	PHQ-9	
Titov et al. (2009) [48*]	Australia	n = 48, telephone interview with MINI 5.0.0	GAD	non-specified assessor	An Internet-based CaCCBT programme for GAD, the Worry programme. 6 online lessons over 9 weeks	individual-guided	GAD-7	PHQ-9	PSWQ

*Abbreviations*: *BAI* Beck Anxiety Inventory, *BDI* Beck Depression Inventory, *BDI-II* Beck Depression Inventory—second edition, *CaCCBT* clinician-assisted CCBT, *CBM* cognitive bias modification, *CBT* cognitive behavioural therapy, *CCBT* computerised cognitive behaviour therapy, *GAD-7* the 7-Item General Anxiety Disorder Scale, *GAD-Q-IV* Generalised Anxiety Disorder Questionnaire-iv, *GAI* Geriatric Anxiety Inventory, *GDS* Geriatric Depression Scale, *HAM-A* Hamilton Anxiety Rating Scale, *HD-ABM* home-delivered attentional bias modification, *ICBT* Internet-based cognitive behavioural therapy, *MADRS-S* Montgomery–Åsberg Depression Rating Scale, *MINI* Mini-International Neuropsychiatric Interview, *PHQ-9* Patient Health Questionnaire-9, *PROMIS* Patient-Reported Outcomes Measurement Information System, *PSWQ* Penn State Worry Questionnaire, *PSWQ-A* Penn State Worry Questionnaire-Abbreviated, *STAI* State-Trait Anxiety Inventory, *STAI-S* State-Trait Anxiety Inventory-State, *STAI-T* State-Trait Anxiety Inventory-Trait

For the meta-analysis focusing on anxiety outcomes, effects assessed by any of the following measures were reported: (i) the 7-item General Anxiety Disorder Scale (GAD-7) [49]; (ii) Beck Anxiety Inventory (BAI) [50]; (iii) the anxiety subscale of the Patient-Reported Outcomes Measurement Information System (PROMIS) [51]; (iv) the Hamilton Anxiety Rating Scale (HAM-A) [52]; (v) Generalised Anxiety Disorder Questionnaire-IV (GAD-Q-IV) [53]; (vi) the State-Trait Anxiety Inventory (STAI) [54]; and (vii) the Geriatric Anxiety Inventory (GAI) [55]. If a study adopted more than one measures for any of the outcomes, we selected the effect based on one scale only, according to the above order of priority (e.g. GAD-7 over BAI, then PROMIS).

For the meta-analysis focusing on depression, effects assessed by any of the following measures were reported: (i) Patient Health Questionnaire-9 (PHQ-9) [56]; (ii) Beck Depression Inventory (BDI) [57]; (iii) Beck Depression Inventory-Second Edition (BDI-II) [58]; (iv) the depression subscale of the PROMIS [51]; (v) the Montgomery–Åsberg Depression Rating Scale (MADRS) [59]; and (vi) the Geriatric Depression Scale (GDS) [60].

For the meta-analysis focusing on worry, only the Penn State Worry Questionnaire (PSWQ) [61] or its abbreviated version (PSWQ-A) [62] was included as it is the single commonly used measure for assessing pathological worry.

### Risk of bias assessment

Risk of bias (RoB) was assessed using the Revised Cochrane risk-of-bias tool for randomized trials (RoB2) [63]. Each included study was evaluated by two independent raters (CP and CYC) according to the following domains: randomisation process, deviations from the intended intervention, missing outcome data, measurement of the outcome, and selection of the reported result. The RoB2 domains and criteria are listed in  Additional file 1:Appendix 1. A study would be considered to have a high overall RoB if five or more of the domain criteria were rated as ‘high risk’ or ‘risk unclear’, and hence removed from the analysis. Any disagreements between the two raters regarding the RoB were settled by discussion with the corresponding author.

### Assessment of publication bias across studies

The presence of publication bias was investigated by several means. First, funnel plots [64] that included each study’s standard error against its effect size were created and visually inspected. Second, the Egger’s test [65] of the intercept was conducted to investigate the association between effect size and result precision. Third, the trim-and-fill procedure [66] was conducted to estimate effect size changes when ‘missing’ small studies were added to the meta-analysis.

### Data synthesis and analysis

This study adopted a meta-analytic approach detailed in [67]. Statistical analysis was conducted using the Comprehensive Meta-Analysis Version 3 [68]. Standardised mean differences were calculated using the means and standard deviations (SD) of each outcome measure at the pre- and post-treatment assessment for both of the treatment and control conditions. We used a random-effects model with 95% confidence intervals to account for variability in sample and methodological characteristics. For each outcome construct (anxiety, depression, and worry), an aggregated effect size (Hedges’ *g*) was calculated to investigate the overall effectiveness of LICBT over the control condition. A Hedges’ *g* of 0.20, 0.50 and 0.80 represents small, moderate, and large effect sizes respectively.

Statistical heterogeneity of effect sizes was evaluated using the tau (T) statistic and the prediction interval. According to Borenstein et al. [69], T is the estimated standard deviation of the true effect sizes for the outcome, whereas the 95% prediction interval represents the range in which the point estimate of 95% of future studies will fall, assuming that true effect sizes are normally distributed [70]. Where significant heterogeneity was reported, it was investigated by meta-regression and sensitivity analysis [71].

## Results

As shown in Fig. 1, a total of 12 studies fulfilled the selection criteria and were included in the meta-analyses. The numbers of studies included in the three meta-analyses were as follows: anxiety (*k* = 12), depression (*k* = 11), and worry (*k* = 9).

### Risk of bias assessment

**Fig. 2:**
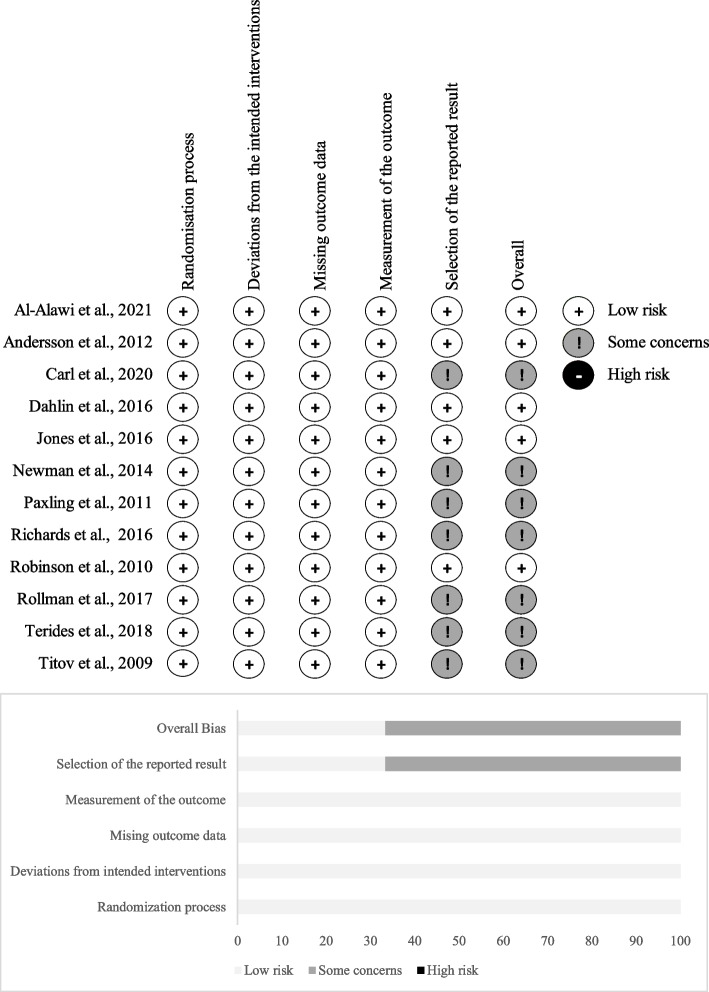
Risk of bias of included studies

The RoB assessment results for all included studies are detailed in Additional file 1: Appendix 2 and summarised in Fig. 2. Seven studies were rated as having ‘some concerns’ on the risk of ‘selection of the reported results’, and hence overall ‘some concerns’ risk ratings were given. These studies either did not have published protocols, or did not indicate the statistical analysis methods mentioned in the published protocol. As no study was rated as ‘high RoB’, all 12 studies remained in the meta-analyses.

### Meta-analysis on the effect of anxiety following LICBT

**Fig. 3:**
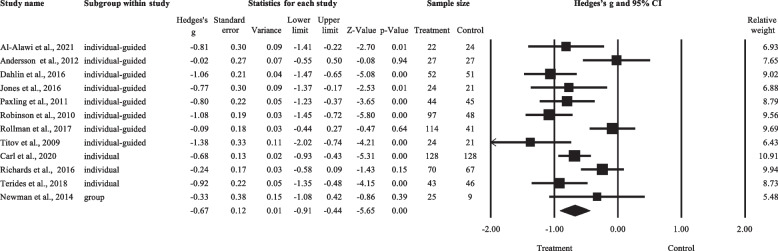
Forest plot for the LICBT effects on anxiety. Abbreviations: individual-guided, individual guided self-help; individual, individual non-facilitated self-help; group, psychoeducational groups

As shown in Fig. 3, a total of 12 effect sizes were included in this meta-analysis, involving a total sample size of 1201. The numbers of studies that tested the three types of LICBT were as follows: individual non-facilitated self-help (*k* = 3), individual guided self-help (*k* = 8), and psychoeducational groups (*k* = 1). The comparison conditions included Internet psychodynamic therapy (*k* = 1), HICBT group (*k* = 1), waitlist condition (*k* = 9), and treatment as usual (TAU, *k* = 1). Anxiety was assessed by using the GAD-7 (*k* = 8), BAI (*k* = 2), PROMIS (*k* = 1), and HARS (*k* = 1). Levels of anxiety for each study are listed in Additional file 1: Appendix 3.

The overall Hedges’ g was -0.67 (95%CI: -0.91 to -0.44), favouring LICBT over control. There was significant between-study heterogeneity (T = 0.34, *I*^2^ = 71.20%, *p* < 0.001), with a 95% prediction interval from -1.47 to 0.13. Subgroup analysis was conducted for subgroups that consisted of at least three effect sizes. The mean effect size of individual-guided self-help studies (k = 8) was -0.74, whereas that of individual-unguided self-help studies (k = 3) was -0.61, both favouring LICBT over control. There was no significant difference in effect size between the two subgroups (Q = 0.21, df = 1, *p* = 0.646). Sensitivity analysis using the ‘leave-one-out’ method did not identify any studies to be removed. Moderation analysis revealed that, across all included studies, baseline level of anxiety did not significantly predict the treatment effect of LICBT (*t* = 2.17, β = 0.57, *p* = 0.055).

**Fig. 4:**
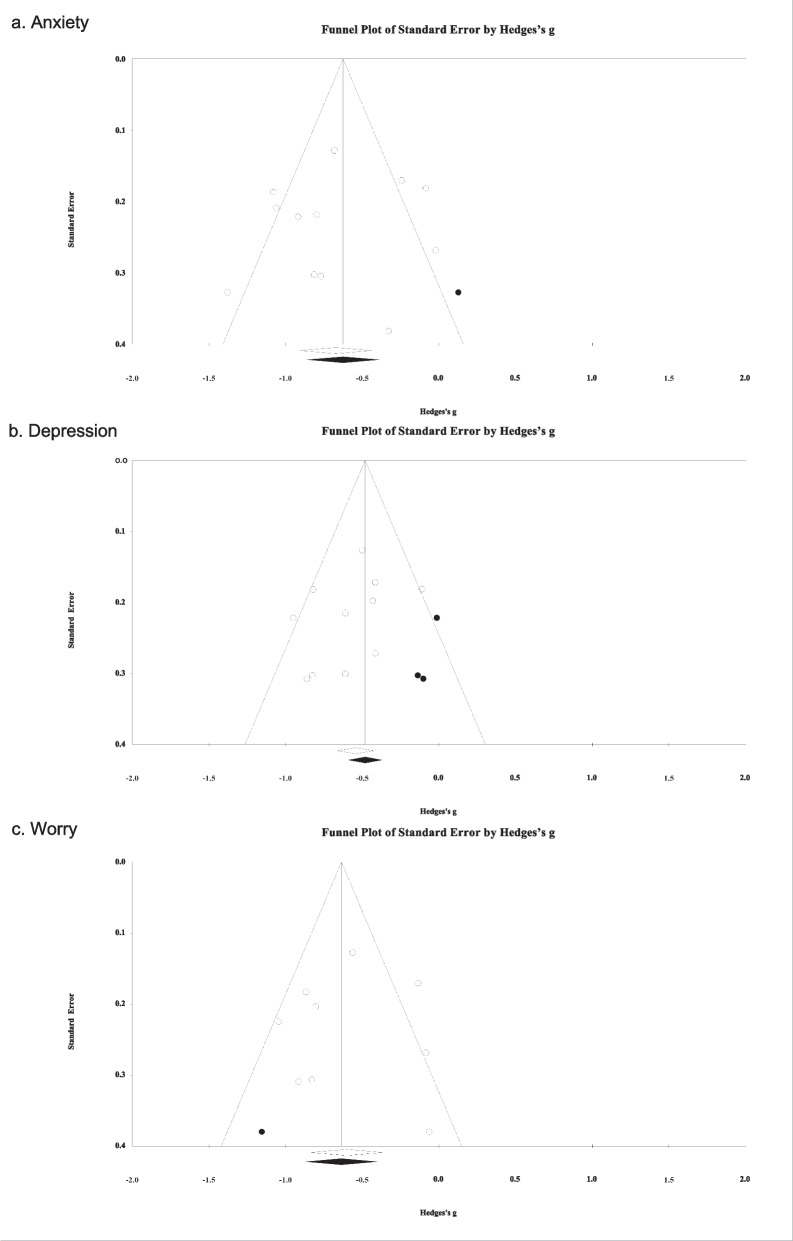
Funnel plots for Hedges’ *gs* on the three outcomes. **a**. Anxiety. **b**. Depression. **c**. Worry

As shown in Fig. 4a, one study would have been trimmed and filled to the right side of the mean for a symmetrical plot. The adjusted effect size (*g* = -0.63) remained significant after imputation (95%CI = -0.86 to -0.39). The Egger’s regression intercept was -0.83 (*p* = 0.668), suggesting no small-study effects.

### Meta-analysis on the effect of depression following LICBT

**Fig. 5:**
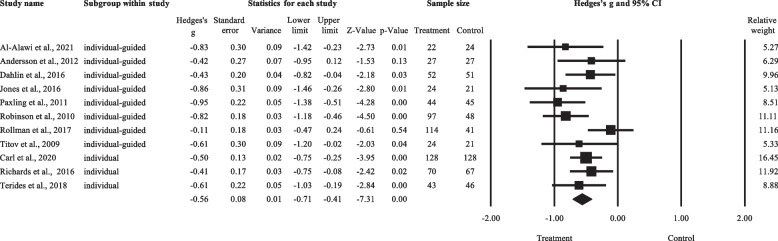
Forest plot for the LICBT effects on depression. Abbreviations: individual-guided, individual guided self-help; individual, individual non-facilitated self-help

As shown in Fig. 5, a total of 11 effect sizes were included in this meta-analysis, involving a total sample size of 1164. The numbers of studies that tested the three types of LICBT were as follows: individual non-facilitated self-help (*k* = 3) and individual guided self-help (*k* = 8). The comparison conditions included Internet psychodynamic therapy (*k* = 1), waitlist condition (*k* = 9), and TAU (*k* = 1). Depression was assessed by using the PHQ-9 (*k* = 7), BDI-II (*k* = 2), BDI (*k* = 1), and PROMIS (*k* = 1). Levels of depression for each study are listed in Additional file 1: Appendix 4.

The overall Hedges’ *g* was -0.56 (95%CI: -0.71 to -0.41), favouring LICBT over control. Between-study heterogeneity was not significant (T = 0.14, *I*^*2*^ = 31.71%, *p* = 0.146), with a 95% prediction interval from -0.92 to -0.20. As shown in Fig. 4b, three studies would have been trimmed and filled to the right side of the mean for a symmetrical plot. The adjusted effect size (*g* = -0.48) remained significant after imputation (95%CI = -0.64 to -0.33). The Egger’s regression intercept was -1.55 (*p* = 0.270), suggesting no small-study effects.

### Meta-analysis on the effect of worry following LICBT

**Fig. 6:**
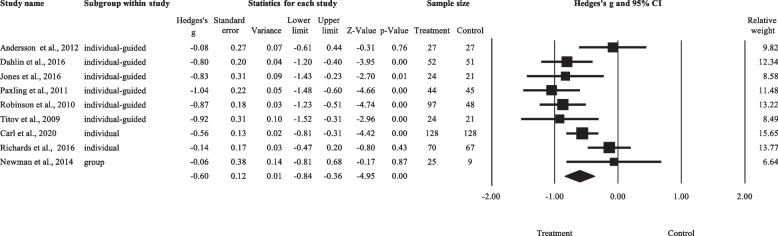
Forest plot for the LICBT effects on worry. Abbreviations: individual-guided, individual guided self-help; individual, individual non-facilitated self-help; group, psychoeducational groups

As shown in Fig. 6, a total of nine effect sizes were included in this meta-analysis, involving a total sample size of 908. The numbers of studies that tested the three types of LICBT were as follows: individual non-facilitated self-help (*k* = 2), individual guided self-help (*k* = 6), and psychoeducational groups (*k* = 1). Comparison conditions included Internet psychodynamic therapy (*k* = 1), typical HICBT group (*k* = 1), and waitlist condition (*k* = 7). Worry was measured by using the PSWQ (*k* = 8) and PSWQ-A (*k* = 1). Levels of worry for each study are listed in Additional file 1: Appendix 5.

The overall Hedges’ *g* was -0.60 (95%CI: -0.84 to -0.36), favouring LICBT over control. There was significant between-study heterogeneity (T = 0.28, *I*^*2*^ = 63.43%, *p* = 0.005), with a 95% prediction interval from -1.32 to 0.12. Since only one intervention subgroup (individual guided self-help) consisted of more than three studies, subgroup analysis was not performed. Sensitivity analysis using the ‘leave-one-out’ method did not identify any studies to be removed. Moderation analysis revealed that, across all included studies, baseline level of worry did not significantly predict the treatment effect of LICBT (*t* = 0.62, β = 0.23, *p* = 0.553).

As shown in Fig. 4c, one study would have been trimmed and filled to the left side of the mean for a symmetrical plot. The adjusted effect size (*g* = -0.64) remained significant after imputation (95%CI = -0.88 to -0.40). The Egger’s regression intercept was -0.22 (*p* = 0.910), suggesting no small-study effects.

## Discussion

This was the first meta-analytic study that systematically integrated the efficacies of the three NICE-recommended LICBT for GAD, namely individual non-facilitated self-help, individual guided self-help, and psychoeducational groups on anxiety, depression, and worry. This study was comprehensive (as an inclusive definition of LICBT was adopted) and stringent (as only randomised controlled trials [RCT] with a low risk of bias were included).

Even though clinical guidelines have suggested LICBT to be first-line psychological intervention for mild to moderate GAD [72], only 12 RCTs that fulfilled our selection criteria were identified over the last 23 years, and the first trial was published in 2009. Four of the trials were conducted in Australia and the UK, where the stepped care model of mental health services was pioneered, whereas the involvement of newer sites (such as Sweden, Canada, USA, and Oman, etc.) reveals that the promise of LICBT has gained traction internationally. Although the development of LICBT for GAD is relatively new, as it has been regularly practised in services worldwide, one would expect that more RCTs would soon emerge in this rapidly growing area.

Overall, LICBT was superior to control conditions in reducing anxiety, depression, and worry, with medium effect sizes (Hedge’s *gs* = -0.67, -0.56, and -0.60). Our first hypothesis was confirmed. For a large majority of the included studies (*k* = 11), the mean scores of the sample fell within the moderate range of anxiety. Although these effect sizes may not be comparable with some of the high-intensity treatment options (e.g. *d* = 0.71–1.67) [73], they are of both statistical and clinical significance. As a briefer and less costly (due to the involvement of para-professionals) intervention than high-intensity options, LICBT potentially maximises the number of beneficiaries within a shorter period of time, constituting an effective and less burdensome alternative for individuals with mild to moderate GAD.

As the three symptoms (anxiety, depression, and worry) are core to the clinical presentation of GAD, it was common for RCTs to report all three outcomes. The fact that effect sizes across outcomes were comparable implies that LICBT is suitable for individuals with GAD as a whole, with or without a depressive comorbidity. It is of note that between-study heterogeneity was significant for two outcomes (anxiety and worry). The levels of heterogeneity (represented by T) in these two analyses were comparable to the average heterogeneity for other meta-analysis (0.33, [74]). None of the moderators tested were significant. Besides, the trim and fill method revealed that even after imputation, the results remained robust, lending support to these aggregated effect sizes as reliable.

Among the 12 included studies, eight involved individual guided self-help, three were non-facilitated self-help, and only one was a psychoeducation group. This reflects a relative lack of attention on the latter two forms of delivery of LICBT, albeit all three were recommended by the NICE guidelines. Effects of all three LICBT subtypes could not be formally compared for all outcomes due to small subgroup sizes. However, it appears that both individual guided self-help and non-facilitated self-help yielded similarly dispersed effect sizes (from small to large) on each of the outcomes, and exploratory subgroup analysis revealed that these two subtypes were not significantly different in effect size on anxiety. In contrast, psychoeducational groups resulted in trivial effect sizes on both outcomes available. Based on the preliminary findings, our hypothesis that *both* individual guided self-help *and* psychoeducational groups will be more efficacious than non-facilitated self-help was not supported.

Our array of included studies encompassed a diverse myriad of treatment components. While some of the components address worry and meta-worry specifically (e.g. worry time and acceptance) [38*, 40*, 41*, 48*], others focus on general skills (e.g. applied relaxation and problem solving) [39*, 41*, 42*, 41*, 44*]. While some are more behavioural by nature (e.g. exposure and activity schedule) [37*, 39*, 41*, 45*], others aim to promote knowledge and awareness (e.g. psychoeducation) [44*, 46*] or address beliefs and meta-beliefs (e.g. cognitive restructuring) [37*, 39*, 41*, 42*, 44*, 45*, 46*]. When more intervention trials become available, an important question would be how LICBT with various therapeutic components compare on clinical outcomes for GAD. It would also be interesting to evaluate whether patients with specific characteristics (such as a strong avoidance tendency or comorbid depression) may benefit more from different subtypes or components of LICBT. These clinically relevant questions could be topics for future research.

Interpretations of the current findings should take into consideration the following issues/limitations. Firstly, except for Newman et al. [42*] and Andersson et al. [38*], most of the included studies compared LICBT against a passive control condition (e.g. waitlist or treatment as usual). While it is conceivable that comparisons against an active control condition would yield smaller effects (see Figs. 3 and 6), the total number of studies did not allow for a subgroup analysis based on control conditions. Secondly, three studies relied on clinical cutoff criteria and did not confirm the participant’s diagnosis of GAD with a clinical interview. Thirdly, the small number of studies and imbalanced subgroup sizes have limited the number of meta-regression analyses that could be adequately powered to address heterogeneity [75, 76]. Lastly, longevity of treatment effects was not formally analysed as follow-ups were available in some studies only, which ranged from four weeks [39*] to three years [43*]. Paxling et al. [43*] reported that treatment effects on anxiety, depression, and worry improved or maintained over three years. This is consistent with the review finding that therapeutic effects of HICBT for anxiety disorders sustained and enlarged over time [77]. Future studies with longer follow-up periods can help to address the question of treatment effect sustainability between LICBT and HICBT for GAD.

Against these caveats, this study confirmed that LICBT, as recommended by the NICE guideline, was efficacious for reducing anxiety, depression, and worry among adults with GAD. The promise of LICBT as an effective, efficient, and practical treatment modality is exciting, especially for GAD which potentially runs a chronic course. Despite the encouraging findings, several questions remain unanswered. Future research that examines various types of LICBT for heterogeneous groups of patients with GAD and follow-up assessments will be warranted. These findings will consolidate the design of LICBT for GAD, both as a standalone treatment and as part of the stepped care service model.

### Supplementary Information

**Additional file 1: Appendix 1. **Revised Cochrane risk-of-bias tool for randomised trials (RoB2) domains and criteria. **Appendix 2.** RoB2 evaluation results of each included study. **Appendix 3.** Anxiety outcomes following LICBT vs. control conditions (k = 12). **Appendix 4.** Depression outcomes following LICBT vs. control conditions (k = 11). **Appendix 5.** Worry outcomes following LICBT vs. control conditions (k = 9). 

## Data Availability

The datasets used and/or analysed during the current study are available from the corresponding author on reasonable request.

## Abbreviations

BAI: Beck Anxiety Inventory
BDI: Beck Depression Inventory
BDI-II: Beck Depression Inventory-Second Edition
DSM: Diagnostic and Statistical Manual of Mental Disorders
GAD: Generalised anxiety disorder
GAD-Q-IV: Generalized Anxiety Disorder Questionnaire-IV
GAI: Geriatric Anxiety Inventory
GDS: Geriatric Depression Scale
HI: High-intensity
HAM-A: Hamilton Anxiety Rating Scale
ICD: International Classification of Diseases
LICBT: Low-intensity cognitive behavioural therapy
MADRS: Montgomery–Åsberg Depression Rating Scale
NICE: National Institute for Health and Care Excellence
PHQ-9: Patient Health Questionnaire-9
PRISMA: Preferred Reporting Items for Systematic Reviews and Meta-Analyses
PROMIS: Patient-Reported Outcomes Measurement Information System
PSWQ: Penn State Worry Questionnaire
RCT: Randomised controlled trials
RoB: Risk of bias

## References

[1] Ruscio AM, Hallion LS, Lim CCW, Aguilar-Gaxiola S, Al-Hamzawi A, Alonso J, Andrade LH, Borges G, Bromet EJ, Bunting B (2017). Cross-sectional comparison of the epidemiology of DSM-5 generalized anxiety disorder across the globe. JAMA Psychiat.

[2] Ter Meulen WG, Draisma S, van Hemert AM, Schoevers RA, Kupka RW, Beekman ATF, Penninx B (2021). Depressive and anxiety disorders in concert-a synthesis of findings on comorbidity in the NESDA study. J Affect Disord.

[3] Zbozinek TD, Rose RD, Wolitzky-Taylor KB, Sherbourne C, Sullivan G, Stein MB, Roy-Byrne PP, Craske MG (2012). Diagnostic overlap of generalized anxiety disorder and major depressive disorder in a primary care sample. Depress Anxiety.

[4] Bjornsson AS, Sibrava NJ, Beard C, Moitra E, Weisberg RB, Benitez CI, Keller MB (2014). Two-year course of generalized anxiety disorder, social anxiety disorder, and panic disorder with agoraphobia in a sample of Latino adults. J Consult Clin Psychol.

[5] Sibrava NJ, Beard C, Bjornsson AS, Moitra E, Weisberg RB, Keller MB (2013). Two-year course of generalized anxiety disorder, social anxiety disorder, and panic disorder in a longitudinal sample of African American adults. J Consult Clin Psychol.

[6] Yonkers KA, Dyck IR, Warshaw M, Keller MB (2000). Factors predicting the clinical course of generalised anxiety disorder. Br J Psychiatry.

[7] Rodriguez BF, Weisberg RB, Pagano ME, Bruce SE, Spencer MA, Culpepper L, Keller MB (2006). Characteristics and predictors of full and partial recovery from generalized anxiety disorder in primary care patients. J Nerv Ment Dis.

[8] Tully PJ, Winefield HR, Baker RA, Denollet J, Pedersen SS, Wittert GA, Turnbull DA (2015). Depression, anxiety and major adverse cardiovascular and cerebrovascular events in patients following coronary artery bypass graft surgery: a five year longitudinal cohort study. BioPsychoSoc Med.

[9] Celano CM, Daunis DJ, Lokko HN, Campbell KA, Huffman JC (2016). Anxiety Disorders and Cardiovascular Disease. Curr Psychiat Rep.

[10] Comer JS, Blanco C, Hasin DS, Liu S-M, Grant BF, Turner JB, Olfson M (2011). Health-related quality of life across the anxiety disorders. J Clin Psychiatry.

[11] Romera I, Fernández-Pérez S, Montejo AL, Caballero F, Caballero L, Arbesú JÁ, Delgado-cohen H, Desaiah D, Polavieja PGD, Gilaberte I (2010). Generalized anxiety disorder, with or without co-morbid major depressive disorder, in primary care: prevalence of painful somatic symptoms, functioning and health status. J Affect Disord.

[12] Revicki DA, Travers K, Wyrwich KW, Svedsäter H, Locklear JC, Mattera MS, Sheehan DV, Montgomery S (2012). Humanistic and economic burden of generalized anxiety disorder in North America and Europe. J Affect Disorders.

[13] Zhu B, Zhao Z, Ye W, Marciniak MD, Swindle RWJ (2009). The cost of comorbid depression and pain for individuals diagnosed with generalized anxiety disorder. J Ner Ment Dis.

[14] Stein MB, Sareen J (2015). Generalized anxiety disorder. New Engl J Med.

[15] National Institute for Health and Care Excellence [NICE]: Common mental health disorders: identification and pathways to care. NICE guideline (CG123) 2011. 31851443

[16] The National Collaborating Centre for Mental Health (2021). Improving Access to Psychological Therapies Manual.

[17] Powell CLYM, Lo AP-K, Yeung GTY, Leung NTY, Mak WWS, So SH-w, et al. A pilot study on the effectiveness of low-intensity cognitive behavioural therapy (LiCBT) for common mental disorders in Hong Kong. Behav Cogn Psychoth. 2021;49(6):758–63. 10.1017/S1352465820000971 33436141

[18] Baigent M, Smith D, Battersby M, Lawn S, Redpath P, McCoy A (2020). The Australian version of IAPT: clinical outcomes of the multi-site cohort study of NewAccess. J Ment Health.

[19] NHS England: Psychological Therapies: reports on the use of IAPT services, England, June 2018 Final, including a report on the IAPT Employment Advisers pilot. In Psychological Therapies, Reports on the use of IAPT services 2018.

[20] National Institute for Health and Care Excellence [NICE]: Generalised anxiety disorder and panic disorder in adults: management. NICE guideline (CG113) 2011. 31961629

[21] LeBlanc NJ, Bartuska AD, Blanchard L, Baker AW, Bui E (2021). Cognitive-behavioral treatment for generalized anxiety disorder: theoretical foundations and empirically supported strategies. Psychiatr Ann.

[22] Newman MG, Zainal NH, Hoyer J (2020). Cognitive-Behavioral Therapy (CBT) for Generalized Anxiety Disorder (GAD). Generalized Anxiety Disorder and Worrying. edn.

[23] Siev J, Chambless DL (2007). Specificity of treatment effects: cognitive therapy and relaxation for generalized anxiety and panic disorders. J Consult Clin Psych.

[24] Dugas MJ, Marchand A, Ladouceur R (2005). Further validation of a cognitive-behavioral model of generalized anxiety disorder: Diagnostic and symptom specificity. J Anxiety Disord.

[25] Covin R, Ouimet AJ, Seeds PM, Dozois DJA (2008). A meta-analysis of CBT for pathological worry among clients with GAD. J Anxiety Disord.

[26] Cuijpers P, Sijbrandij M, Koole SL, Huibers MJH, Berking M, Andersson G (2014). Psychological treatment of generalized anxiety disorder: a meta-analysis. Clin Psychol Rev.

[27] Hanrahan F, Field AP, Jones FW, Davey GCL (2013). A meta-analysis of cognitive therapy for worry in generalized anxiety disorder. Clin Psychol Rev.

[28] Carpenter JK, Andrews LA, Witcraft SM, Powers MB, Smits JA, Hofmann SG (2018). Cognitive behavioral therapy for anxiety and related disorders: A meta-analysis of randomized placebo-controlled trials. Depress Anxiety.

[29] Cromarty P, Drummond A, Francis T, Watson J, Battersby MW (2016). NewAccess for depression and anxiety: adapting the UK Improving Access to Psychological Therapies Program across Australia. Australas Psychiatry.

[30] Shafran R, Myles-Hooton P, Bennett SD, Öst L-G (2021). The concept and definition of low intensity cognitive behaviour therapy. Behav Res Ther.

[31] Eilert N, Enrique Á, Wogan R, Mooney O, Timulak L, Richards D (2020). The effectiveness of Internet-delivered treatment for generalized anxiety disorder: an updated systematic review and meta-analysis. Depress Anxiety.

[32] Richards D, Timulak L, O’Brien E, Hayes C, Viganó N, Sharry J, Doherty G (2015). A randomized controlled trial of an internet-delivered treatment: Its potential as a low-intensity community intervention for adults with symptoms of depression. Eur Psychiat.

[33] Andersson G, Carlbring P, Titov N, Lindefors N (2019). Internet interventions for adults with anxiety and mood disorders: a narrative umbrella review of recent meta-analyses. Can J Psychiatry.

[34] Haug T, Nordgreen T, Öst L-G, Havik OE (2012). Self-help treatment of anxiety disorders: a meta-analysis and meta-regression of effects and potential moderators. Clin Psychol Rev.

[35] Hunot V, Churchill R, de SilvaLima M, Teixeira VB (2007). Psychological therapies for generalised anxiety disorder. Cochrane Database Syst Rev.

[36] Page MJ, McKenzie JE, Bossuyt PM, Boutron I, Hoffmann TC, Mulrow CD, Shamseer L, Tetzlaff JM, Akl EA, Brennan SE (2021). The PRISMA 2020 statement: an updated guideline for reporting systematic reviews. BMJ.

[37] * Al-Alawi M, McCall RK, Sultan A, Al Balushi N, Al-Mahrouqi T, Al Ghailani A, Al Sabti H, Al-Maniri A, Panchatcharam SM, Al Sinawi H. Efficacy of a six-week-long therapist-guided online therapy versus self-help internet-based therapy for COVID-19–induced anxiety and depression: open-label, pragmatic, randomized controlled trial. JMIR Ment Health. 2021;8(2):e26683. 10.2196/26683 PMC7886373 33512323

[38] * Andersson G, Paxling B, Roch-Norlund P, Östman G, Norgren A, Almlöv J, Georén L, Breitholtz E, Dahlin M, Cuijpers P. Internet-based psychodynamic versus cognitive behavioral guided self-help for generalized anxiety disorder: a randomized controlled trial. Psychother Psychosom. 2012;81(6):344–55. 10.1159/000339371 22964540

[39] * Carl JR, Miller CB, Henry AL, Davis ML, Stott R, Smits JA, Emsley R, Gu J, Shin O, Otto MW. Efficacy of digital cognitive behavioral therapy for moderate-to-severe symptoms of generalized anxiety disorder: a randomized controlled trial. Depress Anxiety. 2020;37(12):1168–78. 10.1002/da.23079 32725848

[40] * Dahlin M, Andersson G, Magnusson K, Johansson T, Sjögren J, Håkansson A, Pettersson M, Kadowaki Å, Cuijpers P, Carlbring P. Internet-delivered acceptance-based behaviour therapy for generalized anxiety disorder: a randomized controlled trial. Behav Res Ther. 2016;77:86–95. 10.1016/j.brat.2015.12.007 26731173

[41] * Jones SL, Hadjistavropoulos HD, Soucy JN. A randomized controlled trial of guided internet-delivered cognitive behaviour therapy for older adults with generalized anxiety. J Anx Disord. 2016;37:1–9. 10.1016/j.janxdis.2015.10.006 26561733

[42] * Newman MG, Przeworski A, Consoli AJ, Taylor CB. A randomized controlled trial of ecological momentary intervention plus brief group therapy for generalized anxiety disorder. Psychotherapy. 2014;51(2):198–206. 10.1037/a0032519 PMC4440457 24059730

[43] * Paxling B, Almlöv J, Dahlin M, Carlbring P, Breitholtz E, Eriksson T, Andersson G. Guided Internet-delivered cognitive behavior therapy for generalized anxiety disorder: a randomized controlled trial. Cogn Behav Therapy. 2011;40:159–73. 10.1080/16506073.2011.576699 21770848

[44] * Richards D, Timulak L, Rashleigh C, McLoughlin O, Colla A, Joyce C, Doherty G, Sharry J, Duffy D, Anderson-Gibbons M. Effectiveness of an internet-delivered intervention for generalized anxiety disorder in routine care: a randomised controlled trial in a student population. Internet Interv. 2016;6:80–8. 10.1016/j.invent.2016.10.003 PMC6096214 30135817

[45] * Robinson E, Titov N, Andrews G, McIntyre K, Schwencke G, Solley K. Internet treatment for generalized anxiety disorder: a randomized controlled trial Comparing Clinician vs. Technician Assistance. PLoS One. 2010;5:e10942. 10.1371/journal.pone.0010942 PMC2880592 20532167

[46] * Rollman BL, Herbeck Belnap B, Abebe KZ, Spring MB, Rotondi AJ, Rothenberger SD, Karp JF. Effectiveness of online collaborative care for treating mood and anxiety disorders in primary care. JAMA Psychiatry. 2017;75:56–64. 10.1001/jamapsychiatry.2017.3379 PMC5833533 29117275

[47] * Terides MD, Dear BF, Fogliati VJ, Gandy M, Karin E, Jones MP, et al. Increased skillsusage statistically mediates symptom reduction in self-guided internet-delivered cognitive–behavioural therapy fordepression and anxiety: a randomised controlled trial. Cogn Behav Ther. 2017;47(1):43–61. 10.1080/16506073.2017.1347195. 10.1080/16506073.2017.1347195 28724338

[48] * Titov N, Andrews G, Robinson E, Schwencke G, Johnston L, Solley K, Choi I. Clinician-assisted internet-based treatment is effective for generalized anxiety disorder: randomized controlled trial. Aust NZ J Psychiat. 2009;43:905–12.

[49] Spitzer RL, Kroenke K, Williams JB, Löwe B (2006). A brief measure for assessing generalized anxiety disorder: the GAD-7. Arch Intern Med.

[50] Beck AT, Epstein N, Brown G, Steer RA (1988). An inventory for measuring clinical anxiety: psychometric properties. J Consul Clin Psych.

[51] Cella D, Yount S, Rothrock N, Gershon R, Cook K, Reeve B, Ader D, Fries JF, Bruce B, Rose M (2007). The Patient-Reported Outcomes Measurement Information System (PROMIS): progress of an NIH Roadmap cooperative group during its first two years. Med Care.

[52] Hamilton M (1959). The assessment of anxiety states by rating. Bri J Med Psychol.

[53] Newman MG, Zuellig AR, Kachin KE, Constantino MJ, Przeworski A, Erickson T, Cashman-McGrath L (2002). Preliminary reliability and validity of the Generalized Anxiety Disorder Questionnaire-IV: a revised self-report diagnostic measure of generalized anxiety disorder. Behav Ther.

[54] Spielberger CD, Gorsuch RL, Lushene R, Vagg PR, Jacobs GA (1983). Manual for the State-Trait Anxiety Inventory.

[55] Pachana NA, Byrne GJ, Siddle H, Koloski NA, Harley E, Arnold E (2006). Development and validation of the geriatric anxiety inventory. Int Psychogeriatr.

[56] Kroenke K, Spitzer RL, Williams JB (2001). The PHQ-9: validity of a brief depression severity measure. J Gen Intern Med.

[57] Beck AT, Ward CH, Mendelson M, Mock J, Erbaugh J (1961). An inventory for measuring depression. Arch Gen Psychiat.

[58] Beck AT, Steer RA, Brown G: Beck depression inventory–II. Psychol Assessment 1996.

[59] Montgomery S, Åsberg M (1979). A New Depression Scale Designed to be Sensitive to Change. Bri J Psychiatry.

[60] Sheikh J, Yesavage JA (1986). Geriatric Depression Scale (GDS): Recent evidence and development of a shorter version. Clin Gerontologist.

[61] Meyer TJ, Miller ML, Metzger RL, Borkovec TD (1990). Development and validation of the Penn State Worry Questionnaire. Behav Res Ther.

[62] Hopko DR, Reas DL, Beck JG, Stanley MA, Wetherell JL, Novy DM, Averill PM (2003). Assessing worry in older adults: confirmatory factor analysis of the Penn State Worry Questionnaire and psychometric properties of an abbreviated model. Psychol Assessment.

[63] Sterne JAC, Savović J, Page MJ, Elbers RG, Blencowe NS, Boutron I, Cates CJ, Cheng H-Y, Corbett MS, Eldridge S (2019). RoB 2: a revised tool for assessing risk of bias in randomised trials. BMJ.

[64] Lin L, Chu H (2018). Quantifying publication bias in meta-analysis. Biometrics.

[65] Egger M, Smith GD, Schneider M, Minder C (1997). Bias in meta-analysis detected by a simple, graphical test. BMJ.

[66] Duval S, Tweedie R (2000). Trim and fill: a simple funnel-plot–based method of testing and adjusting for publication bias in meta-analysis. Biometrics.

[67] Cooper H, Hedges LV, Valentine JC: The handbook of research synthesis and meta-analysis: Russell Sage Foundation; 2019.

[68] Borenstein M, Hedges L, Higgins J, Rothstein H (2005). Comprehensive meta-analysis version 3. Englewood, NJ. Biostatistics.

[69] Borenstein M, Hedges L, Higgins J, Rothstein H: Introduction to meta-analysis: John Wiley & Sons; 2009.

[70] Riley RD, Higgins JPT, Deeks JJ (2011). Interpretation of random effects meta-analyses. BMJ.

[71] Viechtbauer W, Cheung MWL (2010). Outlier and influence diagnostics for meta-analysis. Res Synth Methods.

[72] National Collaborating Centre for Mental Health (UK) (2011). Generalised Anxiety Disorder in Adults: Management in Primary, Secondary and Community Care.

[73] van der Heiden C, Muris P, van der Molen HT (2012). Randomized controlled trial on the effectiveness of metacognitive therapy and intolerance-of-uncertainty therapy for generalized anxiety disorder. Behav Res Ther.

[74] Linden AH, Hönekopp J (2021). Heterogeneity of research results: A new perspective from which to assess and promote progress in psychological science. Perspect Psychol Sci.

[75] Burke JF, Sussman JB, Kent DM, Hayward RA (2015). Three simple rules to ensure reasonably credible subgroup analyses. BMJ.

[76] Cuijpers P, Griffin JW, Furukawa TA (2021). The lack of statistical power of subgroup analyses in meta-analyses: a cautionary note. Epidemiol Psychiatr Sci.

[77] Bandelow B, Sagebiel A, Belz M, Görlich Y, Michaelis S, Wedekind D (2018). Enduring effects of psychological treatments for anxiety disorders: meta-analysis of follow-up studies. Bri J Psychiatry.

