# Supplementary material for: A meta-analysis on the efficacy of low-intensity cognitive behavioural therapy for generalised anxiety disorder
Source: BMC Psychiatry. 2024 Jan 2;24:10. doi: 10.1186/s12888-023-05306-6 (PMC10763350; doi:10.1186/s12888-023-05306-6)
Supplement: Supplementary file 1 — Additional file 1: Appendix 1. Revised Cochrane risk-of-bias tool for randomised trials (RoB2) domains and criteria. Appendix 2. RoB2 evaluation results of each included study. Appendix 3. Anxiety outcomes following LICBT vs. control conditions (k = 12). Appendix 4. Depression outcomes following LICBT vs. control conditions (k = 11). Appendix 5. Worry outcomes following LICBT vs. control conditions (k = 9). [file 12888_2023_5306_MOESM1_ESM.docx]

Appendix 1. Revised Cochrane risk-of-bias tool for randomised trials (RoB2) domains and criteria

**Domain 1: Risk of bias arising from the randomisation process**

1.1 Was the allocation sequence random?

1.2 Was the allocation sequence concealed until participants were enrolled and assigned to interventions?

1.3 Did baseline differences between intervention groups suggest a problem with the randomization process?

Optional: What is the predicted direction of bias arising from the randomisation process?

**Domain 2: Risk of bias due to deviations from the intended interventions**

2.1. Were participants aware of their assigned intervention during the trial?

2.2. Were carers and people delivering the interventions aware of participants' assigned intervention during the trial?

2.3. If Y/PY/NI to 2.1 or 2.2: Were there deviations from the intended intervention that arose because of the trial context?

2.4 If Y/PY to 2.3: Were these deviations likely to have affected the outcome?

2.5. If Y/PY/NI to 2.4: Were these deviations from intended intervention balanced between groups?

2.6 Was an appropriate analysis used to estimate the effect of assignment to intervention?

2.7 If N/PN/NI to 2.6: Was there potential for a substantial impact (on the result) of the failure to analyse participants in the group to which they were randomised?

Optional: What is the predicted direction of bias due to deviations from intended interventions?

**Domain 3: Missing outcome data**

3.1 Were data for this outcome available for all, or nearly all, participants randomized?

3.2 If N/PN/NI to 3.1: Is there evidence that the result was not biased by missing outcome data?

3.3 If N/PN to 3.2: Could missingness in the outcome depend on its true value?

3.4 If Y/PY/NI to 3.3: Is it likely that missingness in the outcome depended on its true value?

Optional: What is the predicted direction of bias due to missing outcome data?

**Domain 4: Risk of bias in measurement of the outcome**

4.1 Was the method of measuring the outcome inappropriate?

4.2 Could measurement or ascertainment of the outcome have differed between intervention groups?

4.3 If N/PN/NI to 4.1 and 4.2: Were outcome assessors aware of the intervention received by study participants?

4.4 If Y/PY/NI to 4.3: Could assessment of the outcome have been influenced by knowledge of intervention received?

4.5 If Y/PY/NI to 4.4: Is it likely that assessment of the outcome was influenced by knowledge of intervention received?

Optional: What is the predicted direction of bias in measurement of the outcome?

**Domain 5: Risk of bias in selection of the reported result**

5.1 Were the data that produced this result analysed in accordance with a pre-specified analysis plan that was finalized before unblinded outcome data were available for analysis?

Is the numerical result being assessed likely to have been selected, on the basis of the results, from...

5.2. ... multiple eligible outcome measurements (e.g. scales, definitions, time points) within the outcome domain?

5.3 ... multiple eligible analyses of the data?

Optional: What is the predicted direction of bias due to selection of the reported result?

Appendix 2. RoB2 evaluation results of each included study

Appendix 2.1 RoB2 result of Al-Alawi et al. (2021)


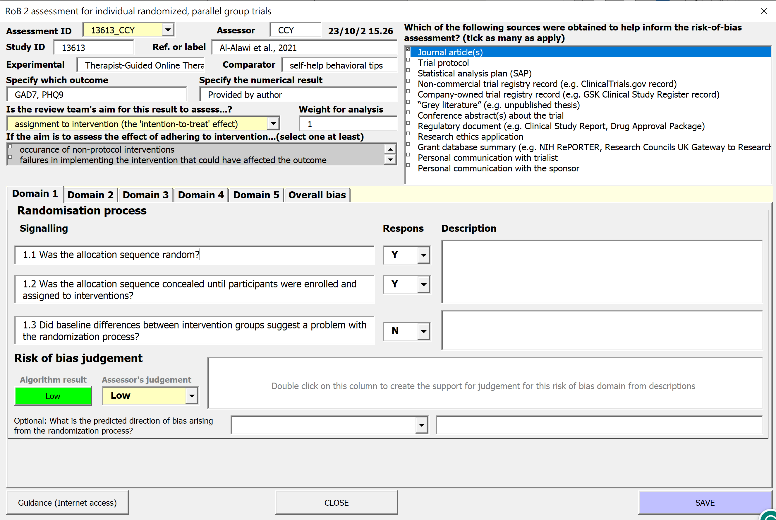

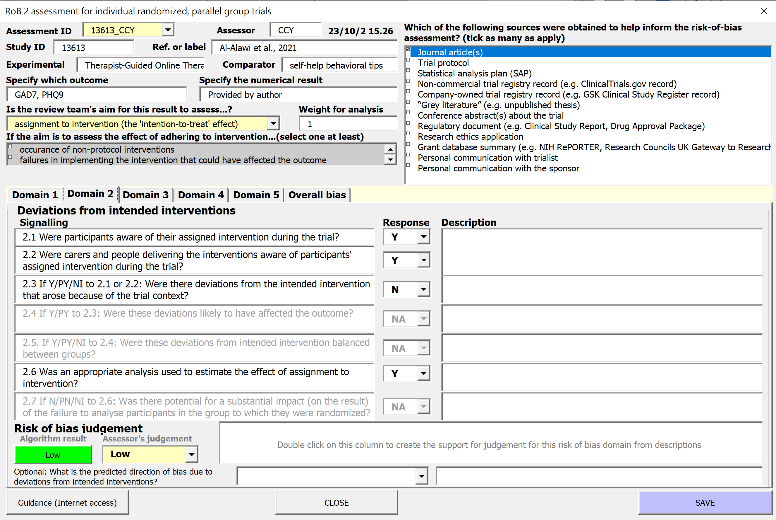

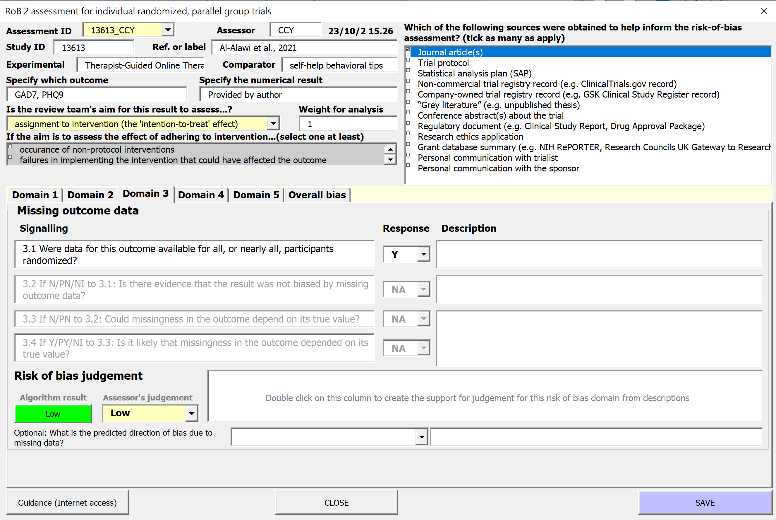

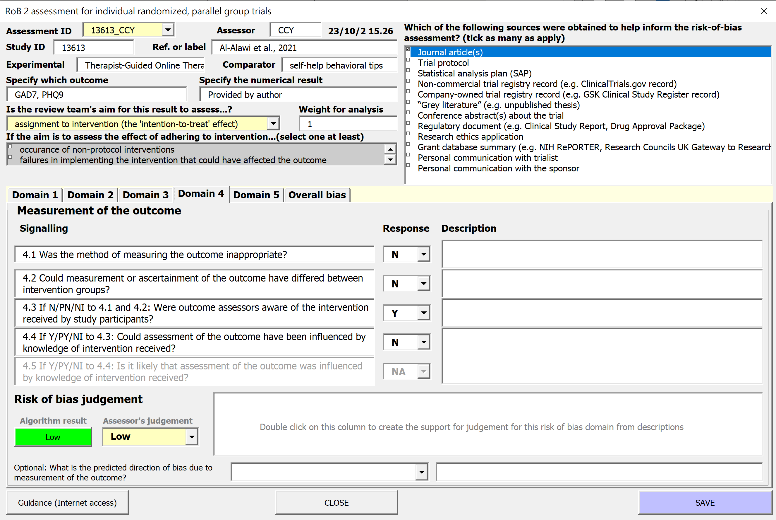

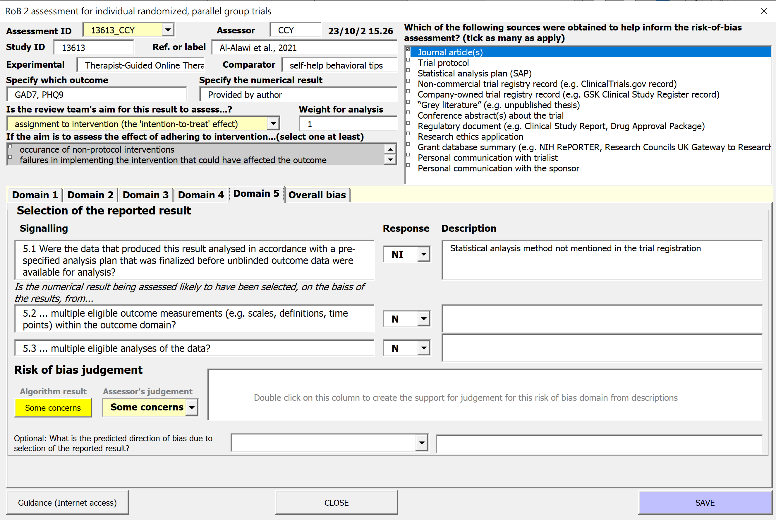

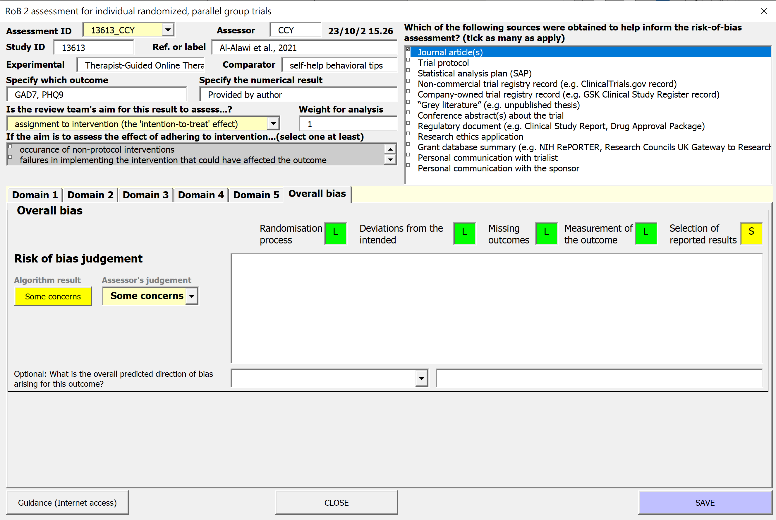


Appendix 2. RoB2 evaluation results of each included study

Appendix 2.1 RoB2 result of Al-Alawi et al. (2021)


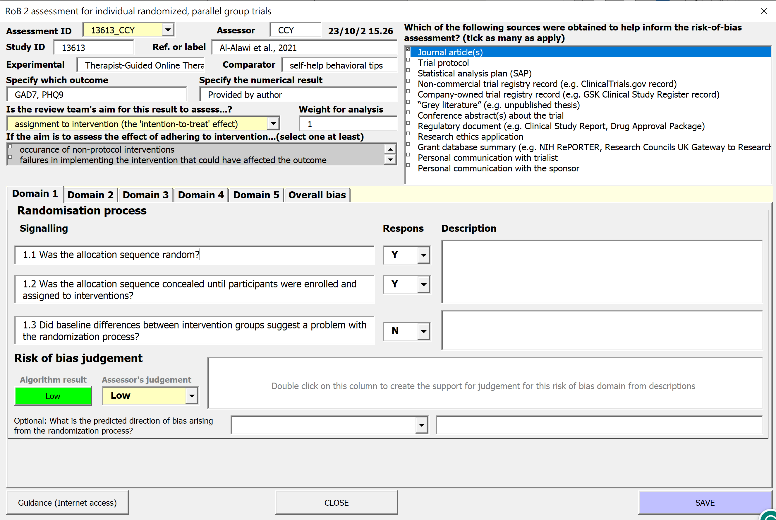

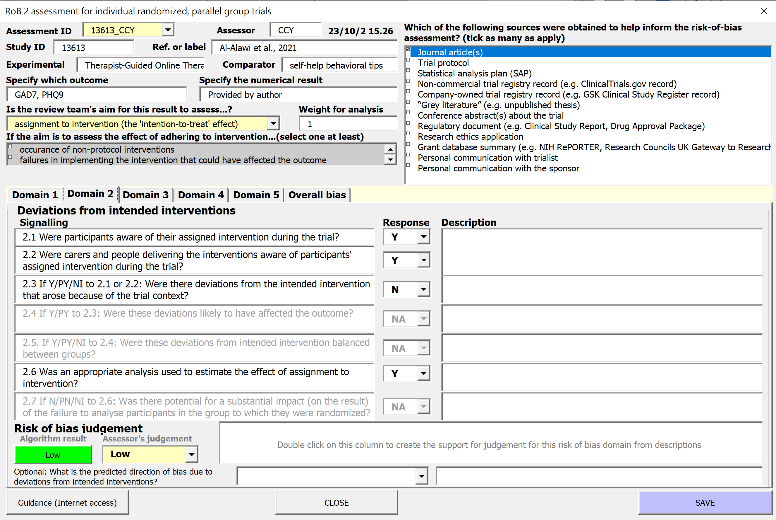

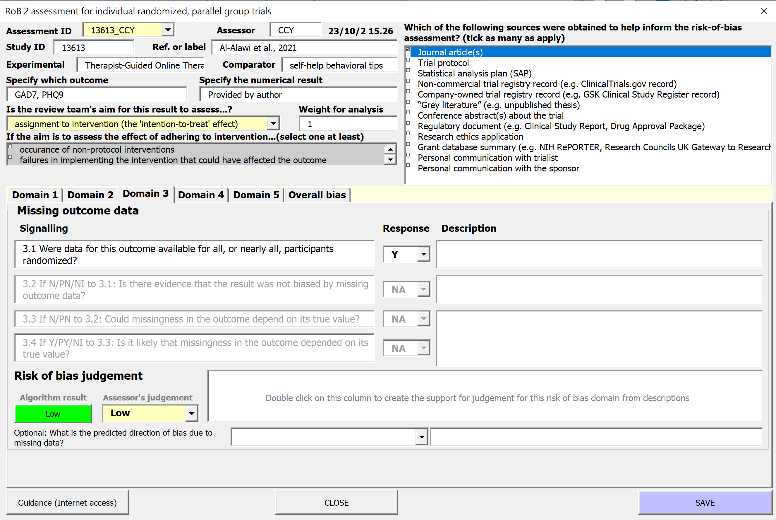

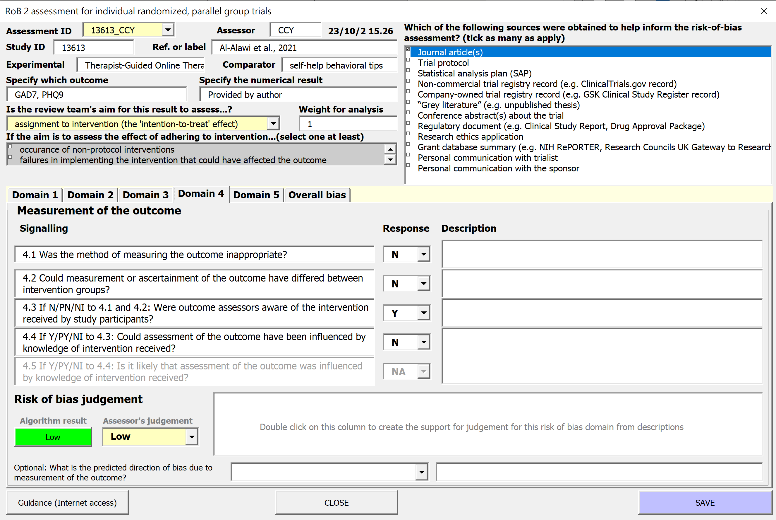

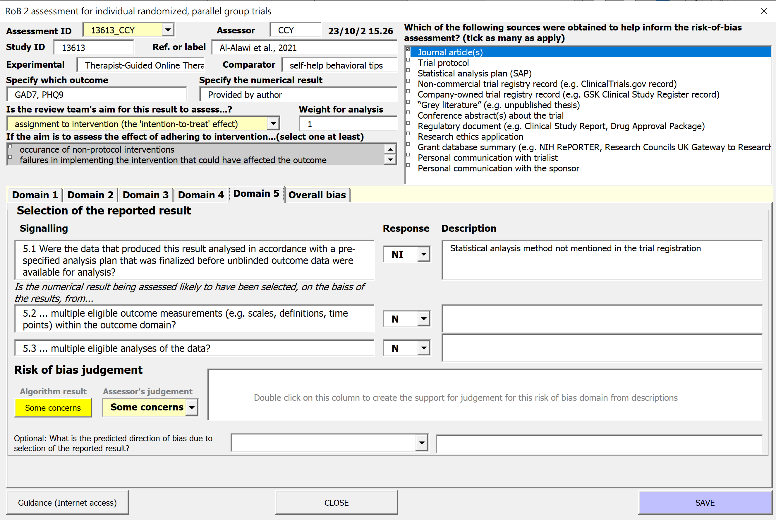

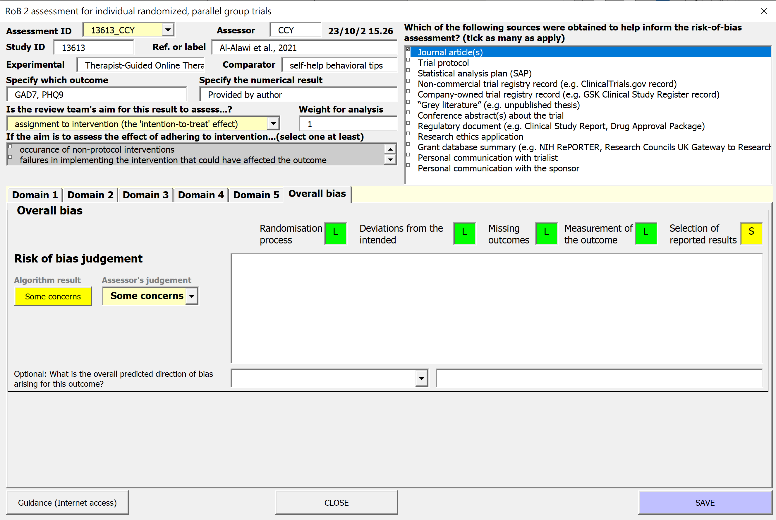


Appendix 2.2 RoB2 result of Andersson et al. (2012)


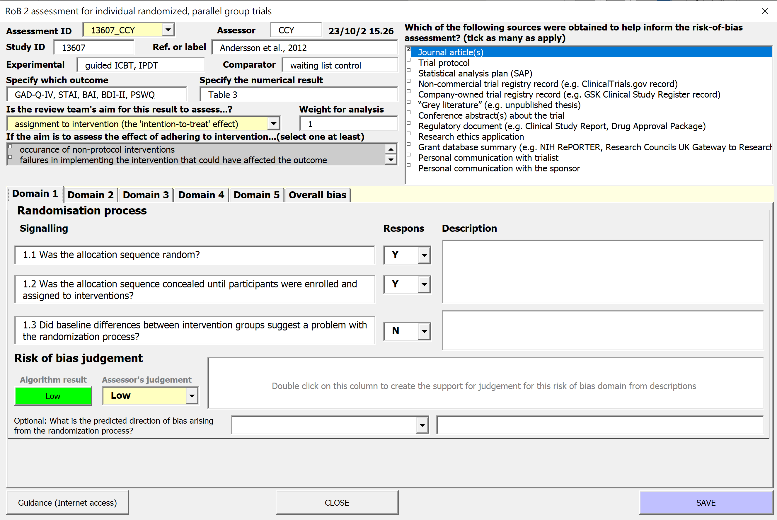

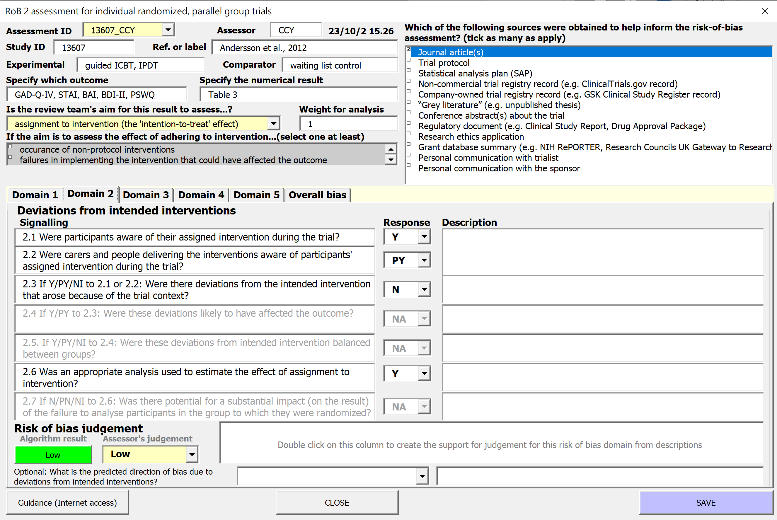

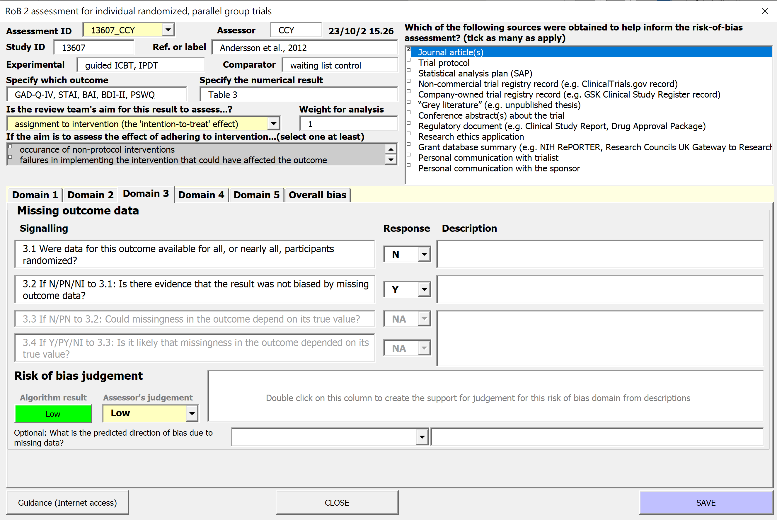

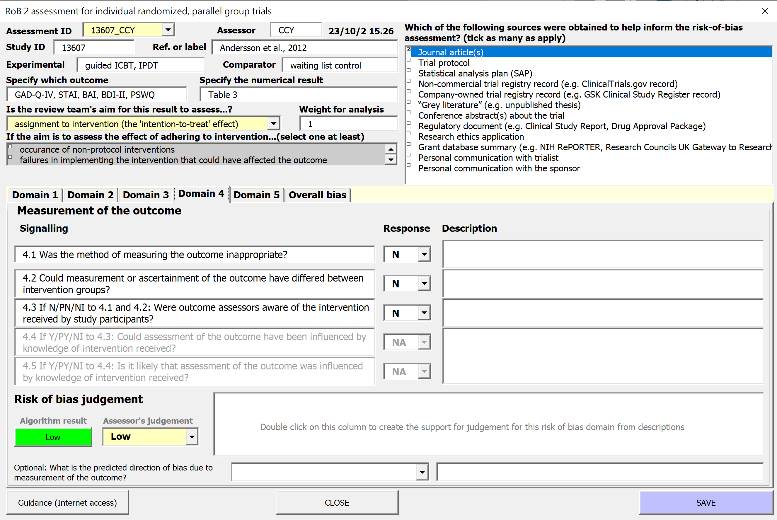

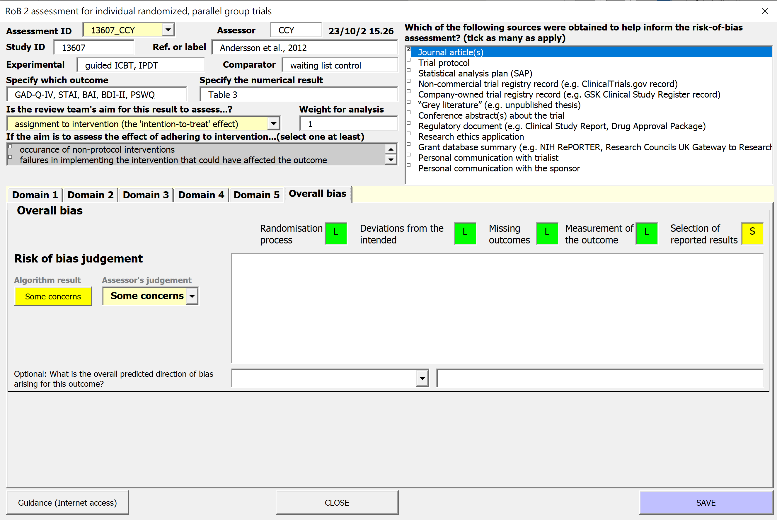

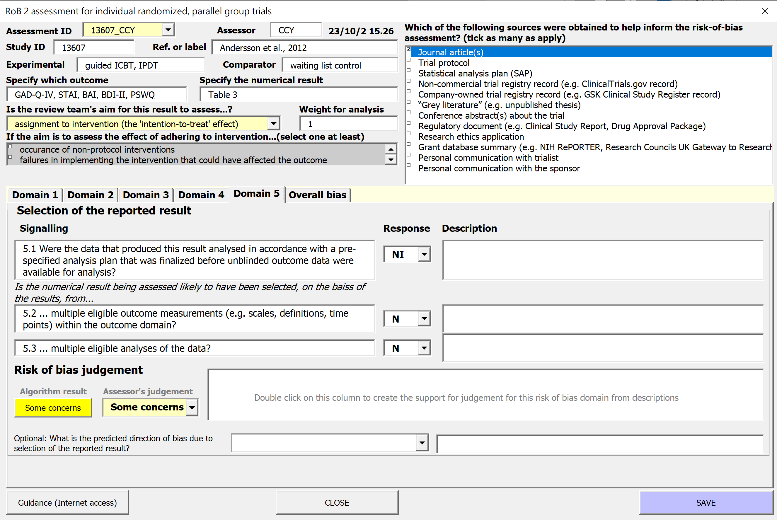


Appendix 2.3 RoB2 result of Carl et al. (2020)


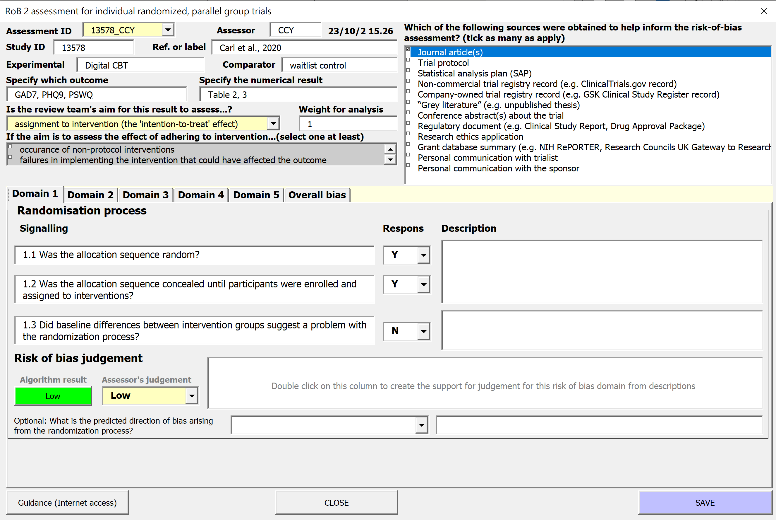

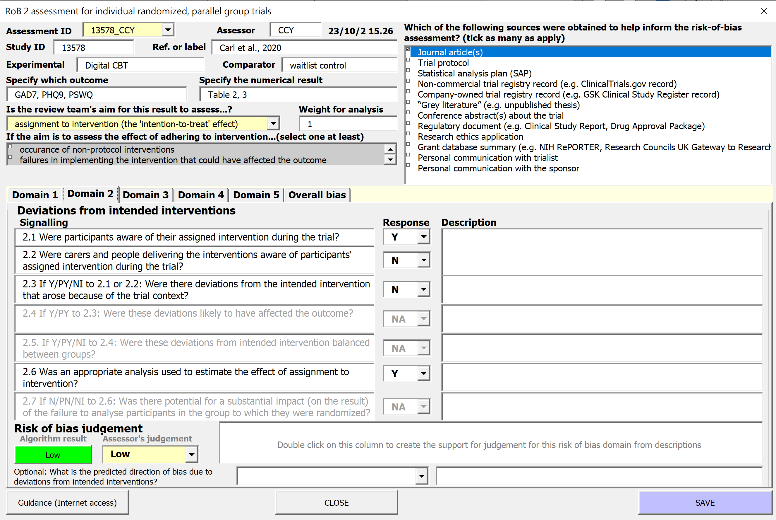

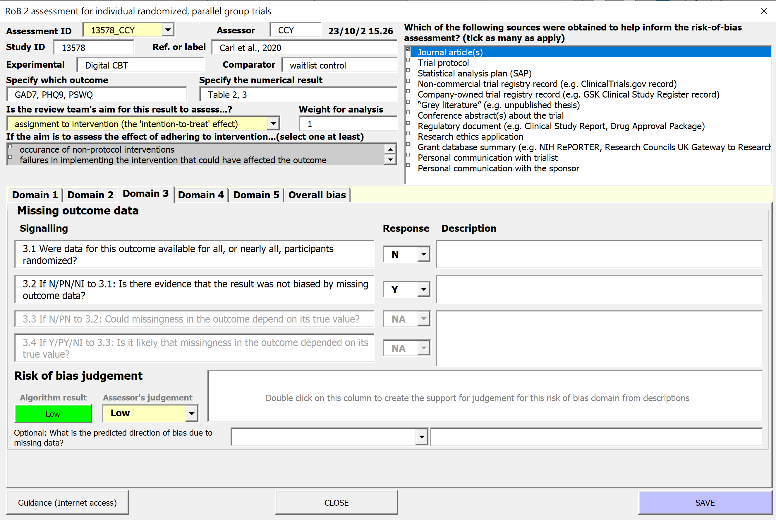

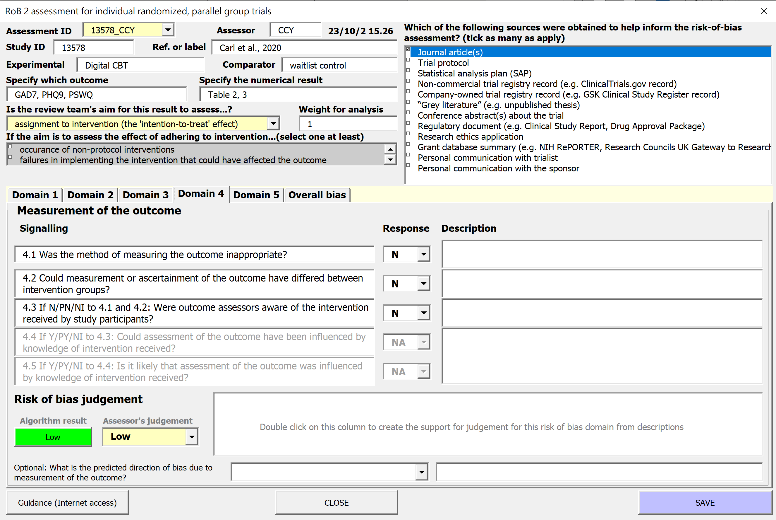

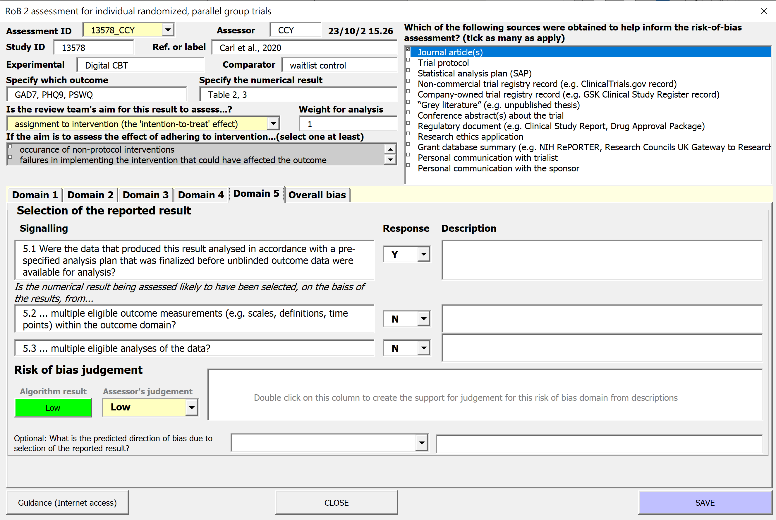

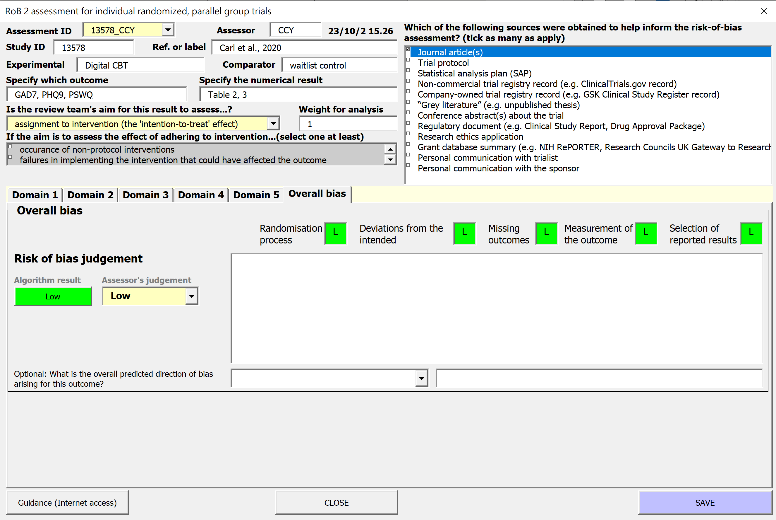


Appendix 2.4 RoB2 result of Dahlin et al. (2016)


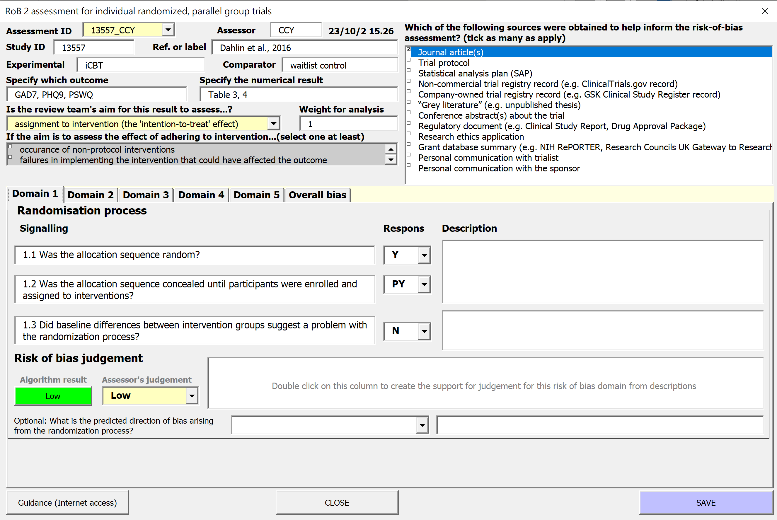

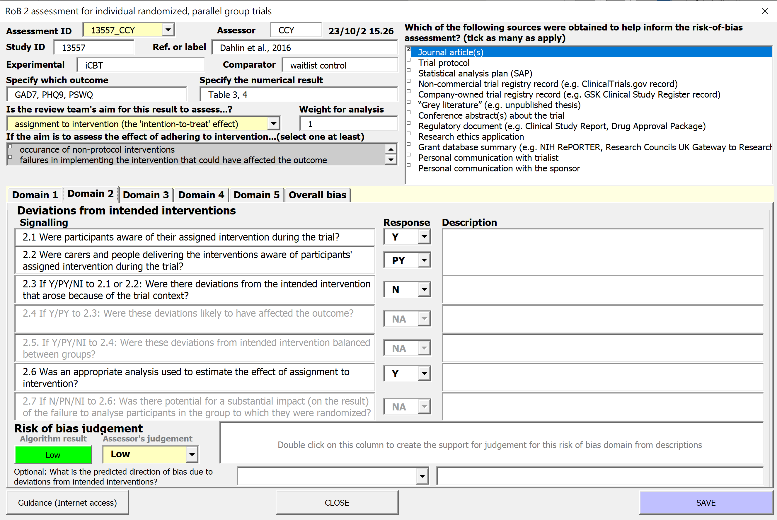

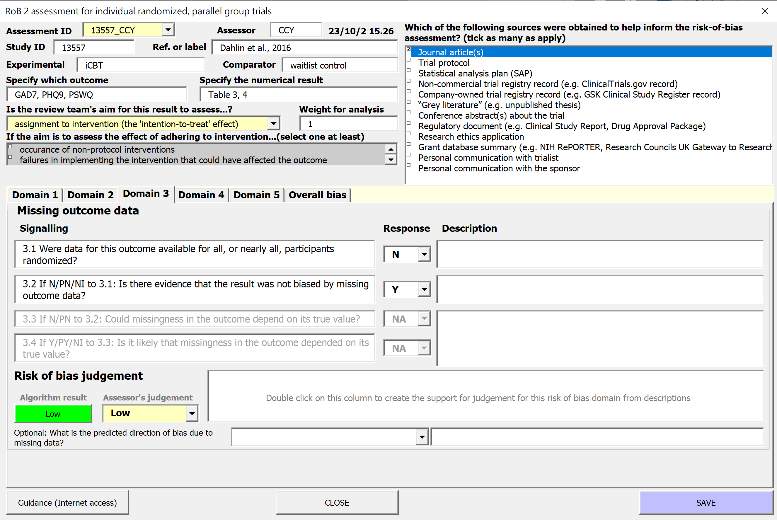

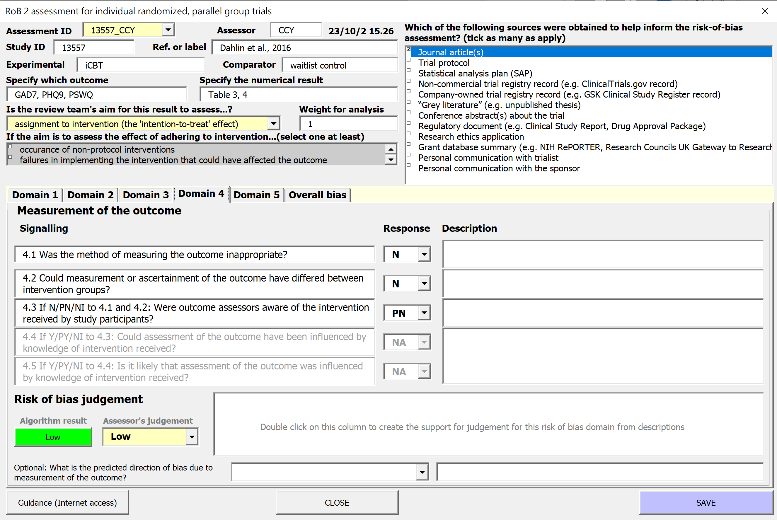

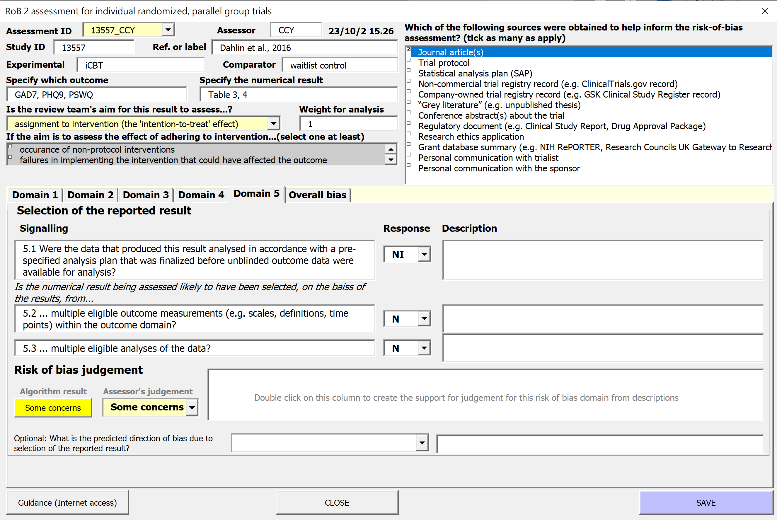

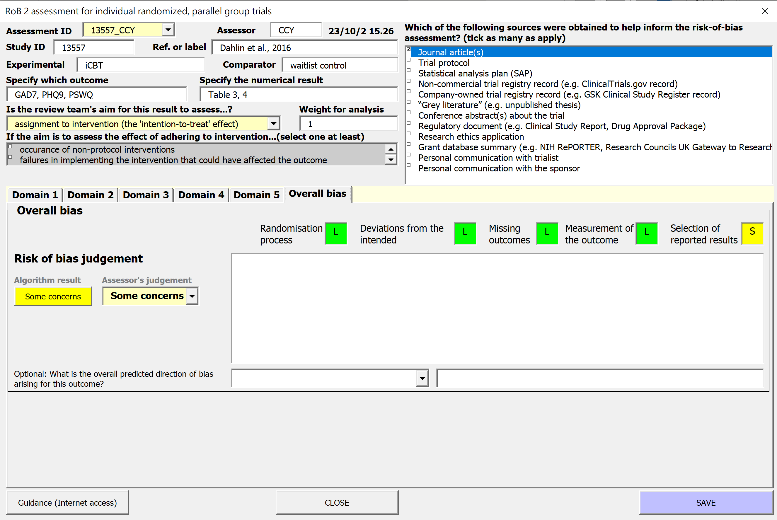


Appendix 2.5 RoB2 result of Jones et al. (2016)


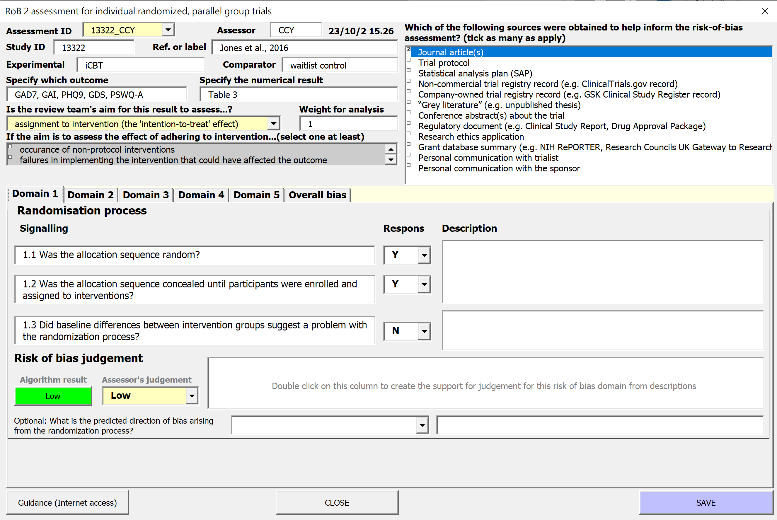

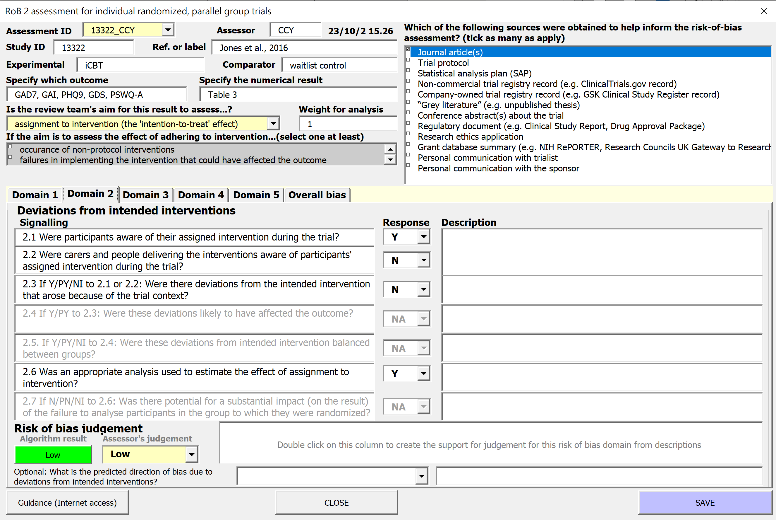

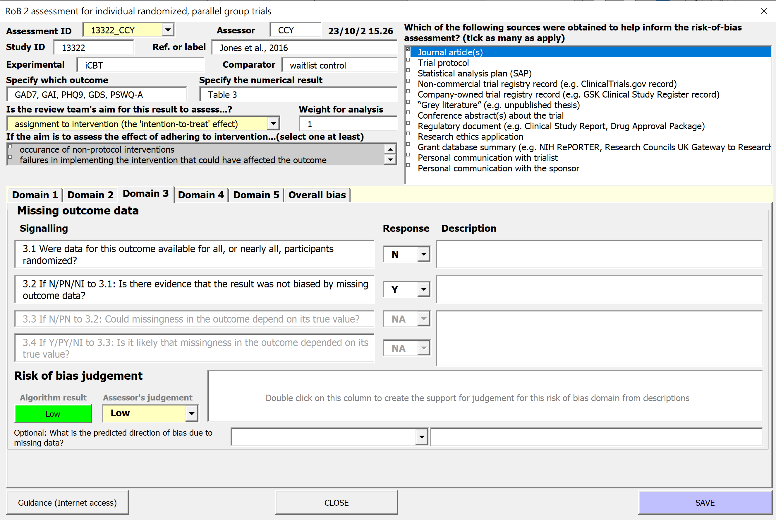

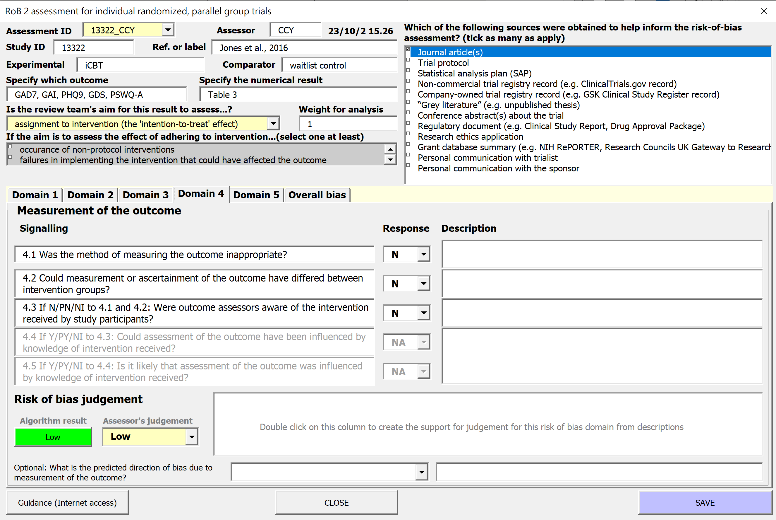

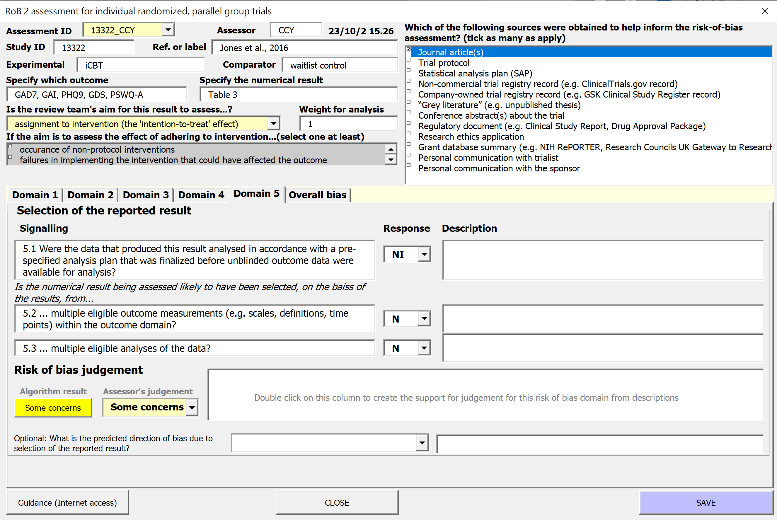

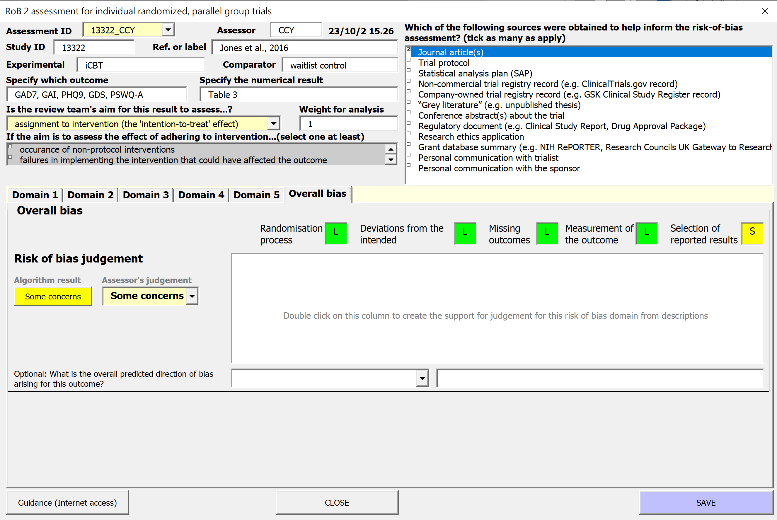


Appendix 2.6 RoB2 result of Newman et al. (2014)


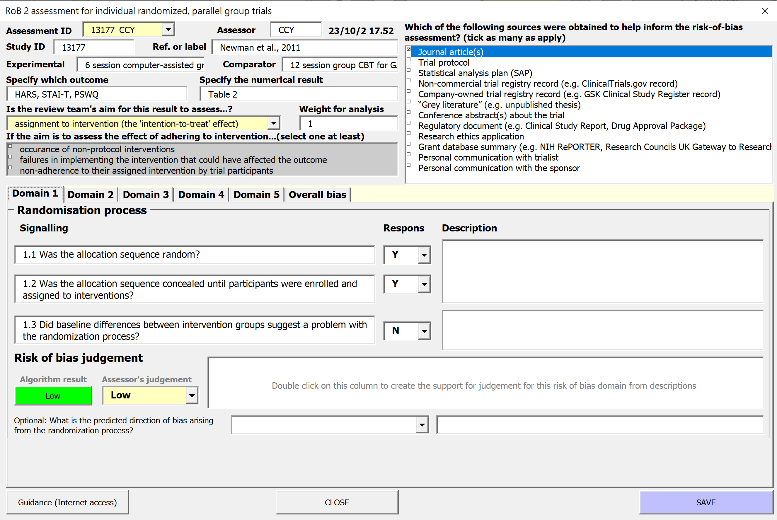

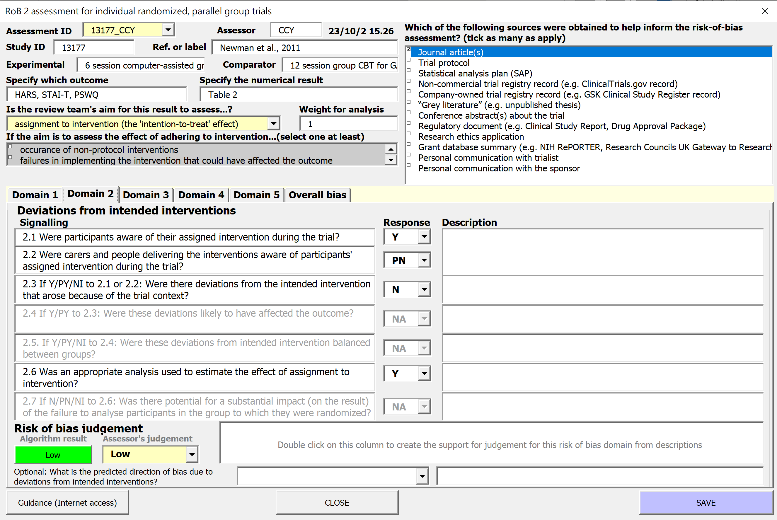

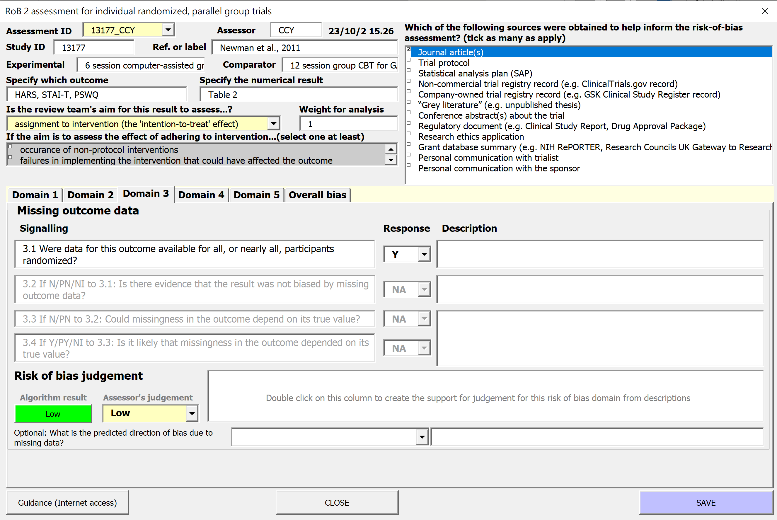

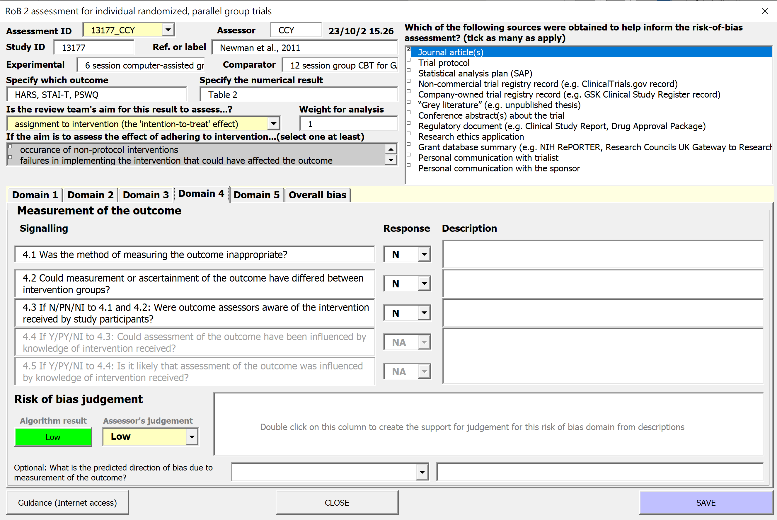

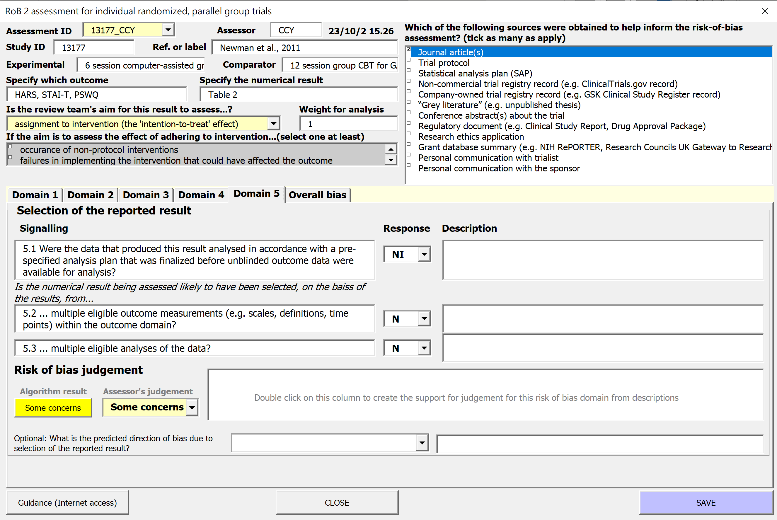

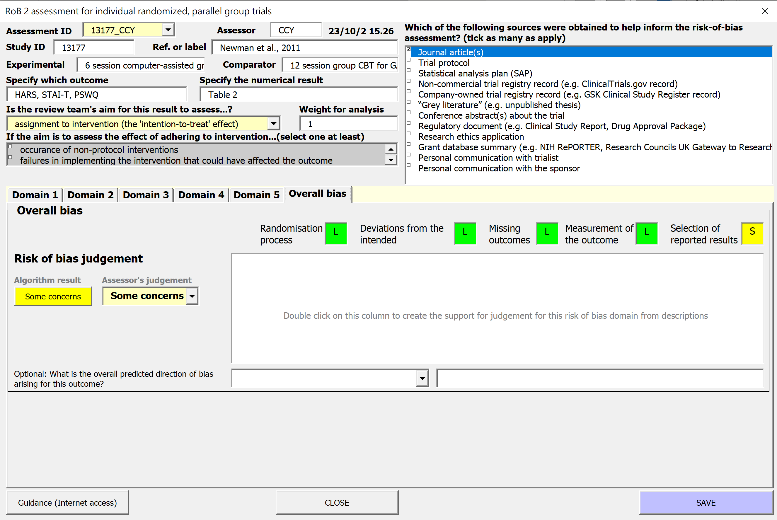


Appendix 2.7 RoB2 result of Paxling et al. (2011)


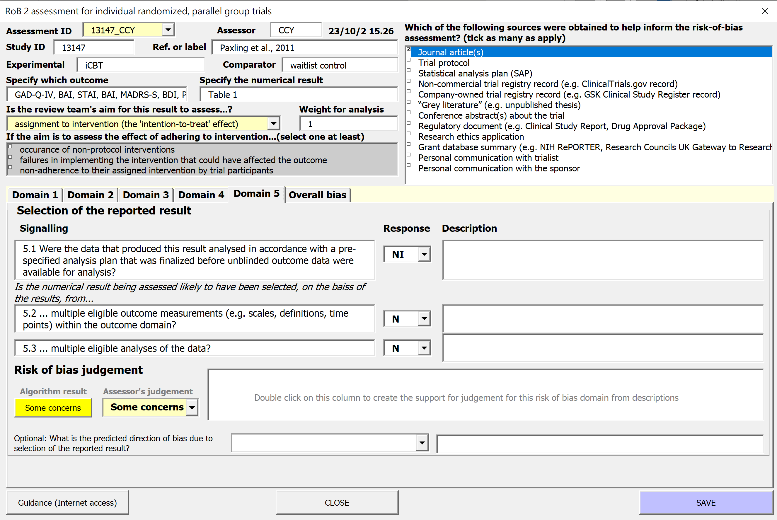

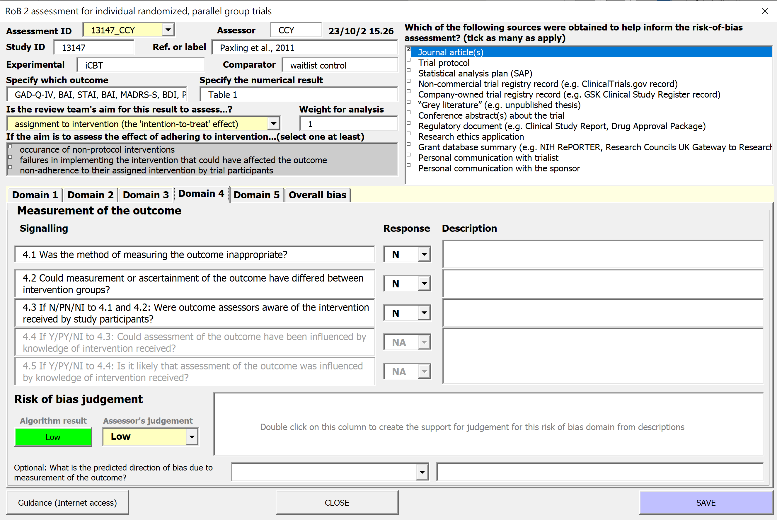

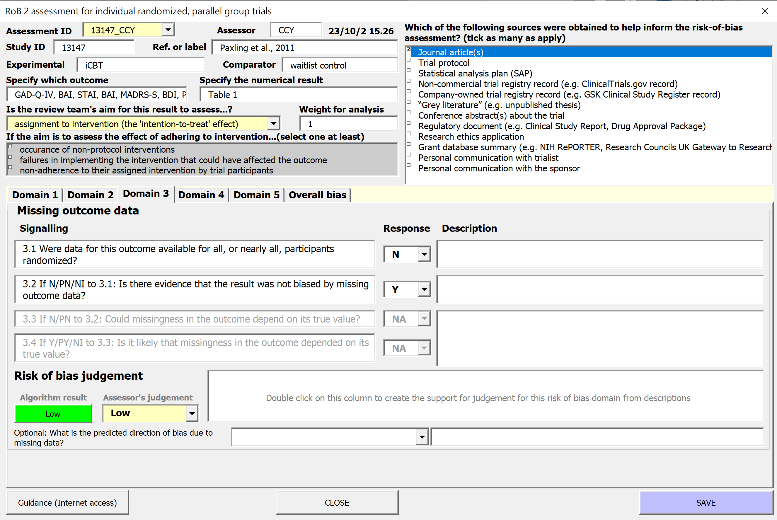

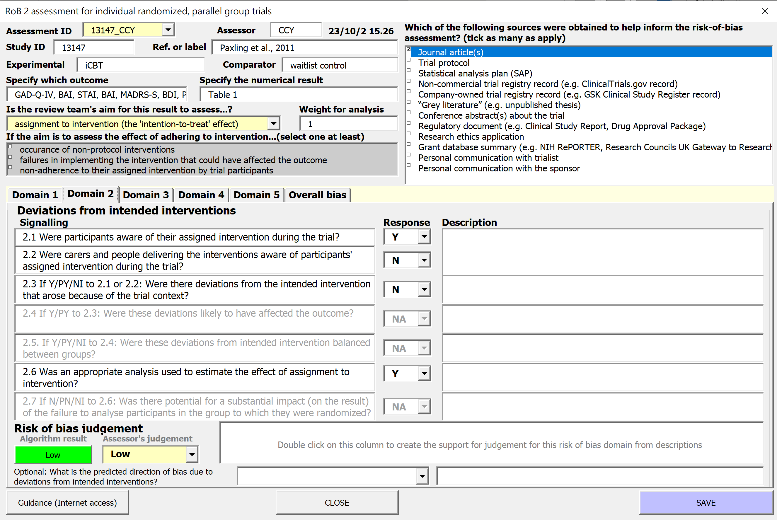

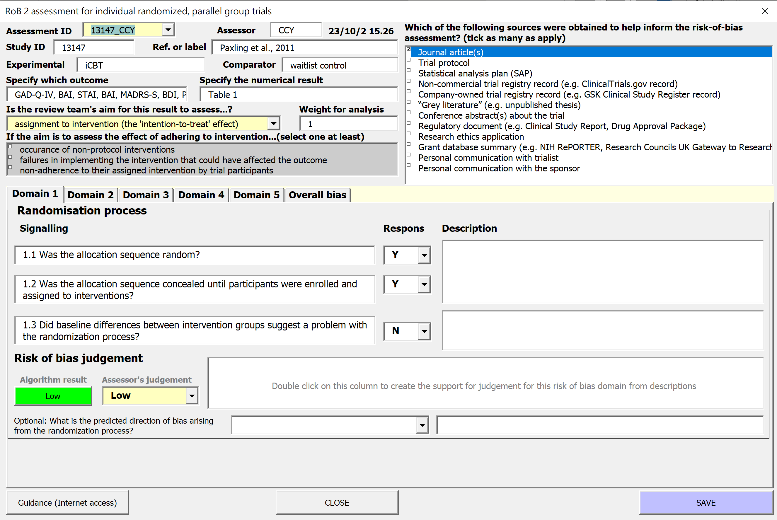

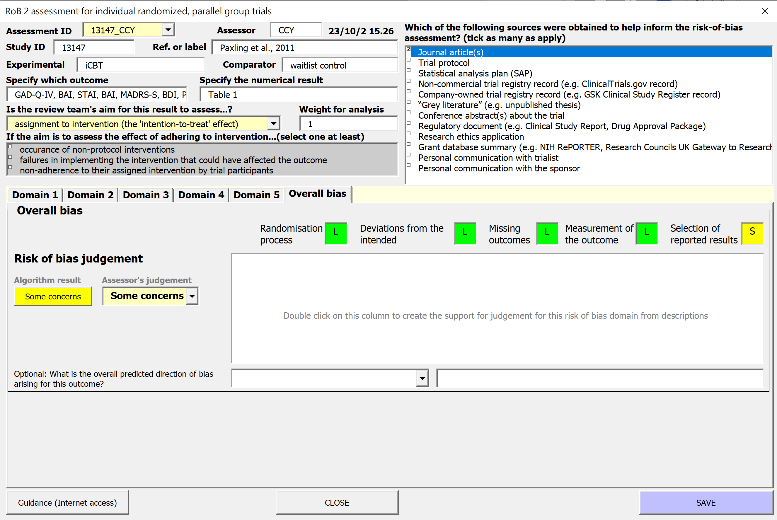


Appendix 2.8 RoB2 result of Richards et al. (2016)


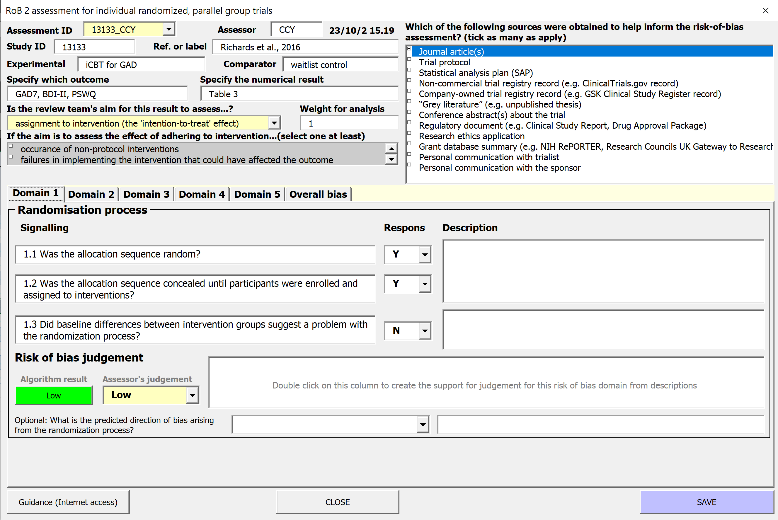

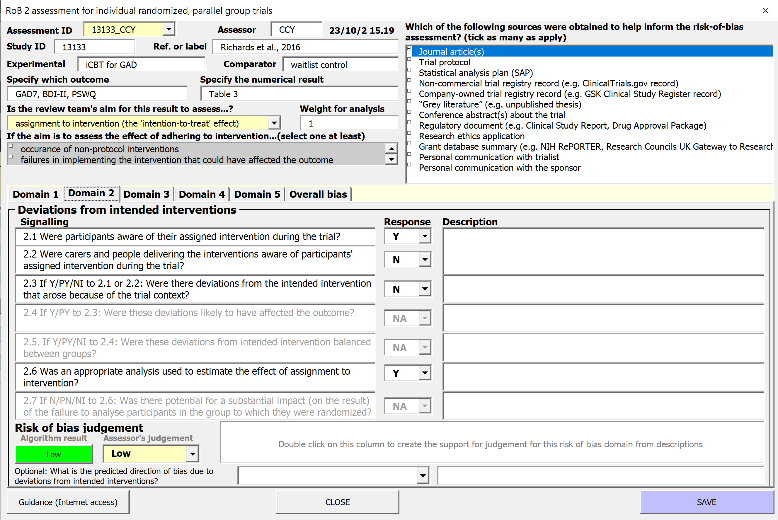

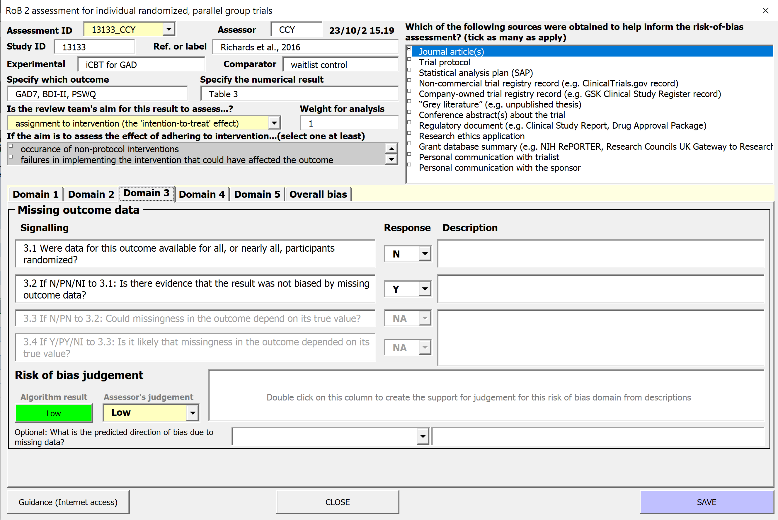

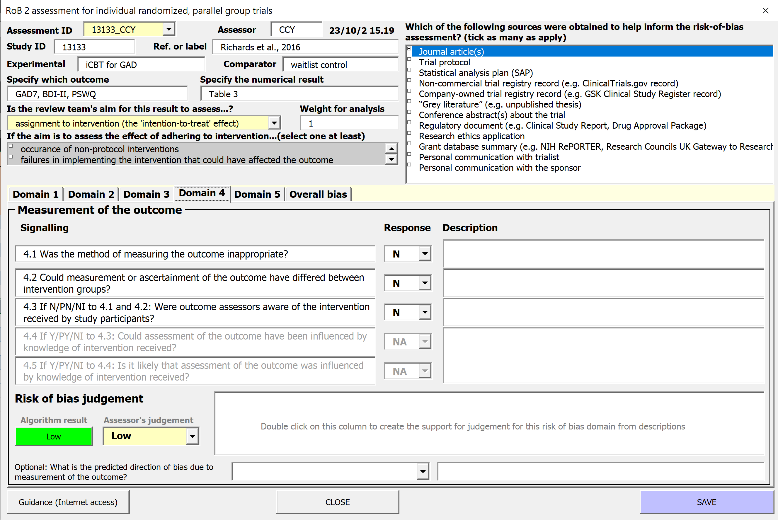

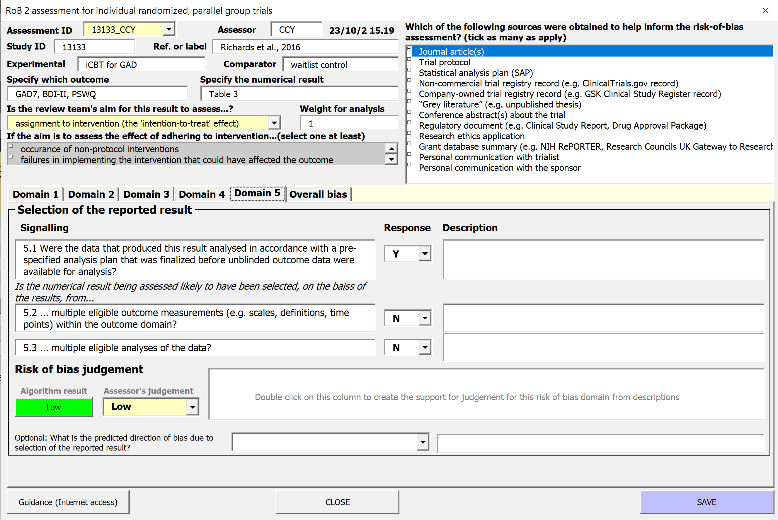

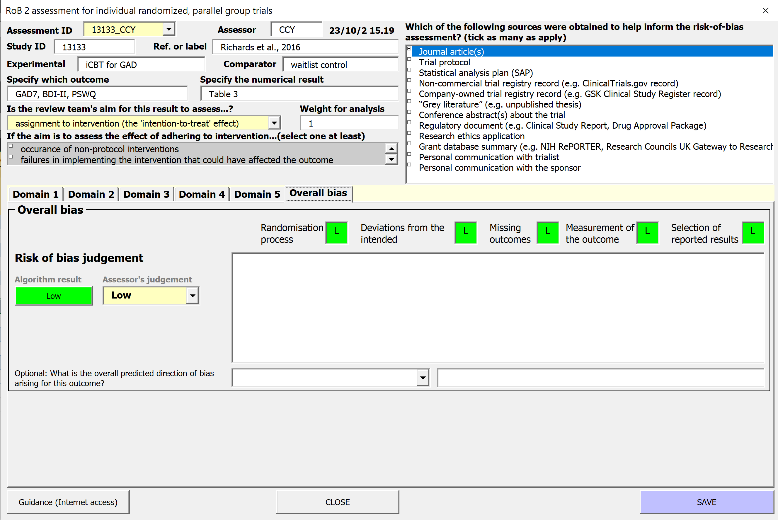


Appendix 2.9 RoB2 result of Robinson et al. (2010)


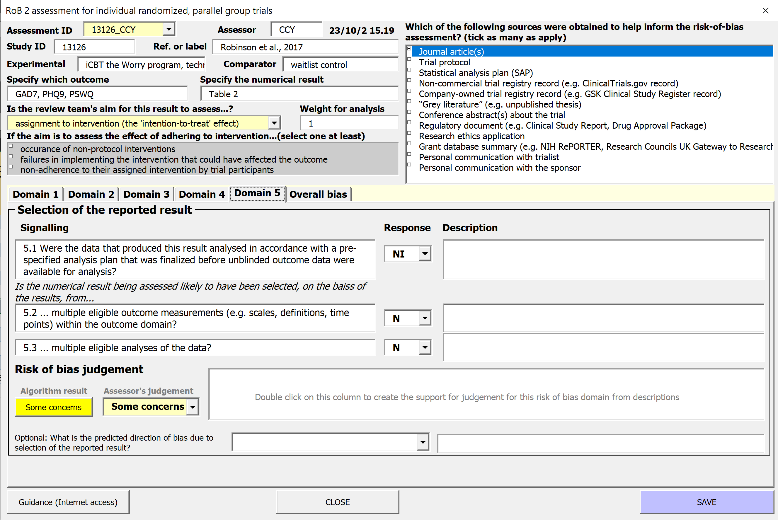

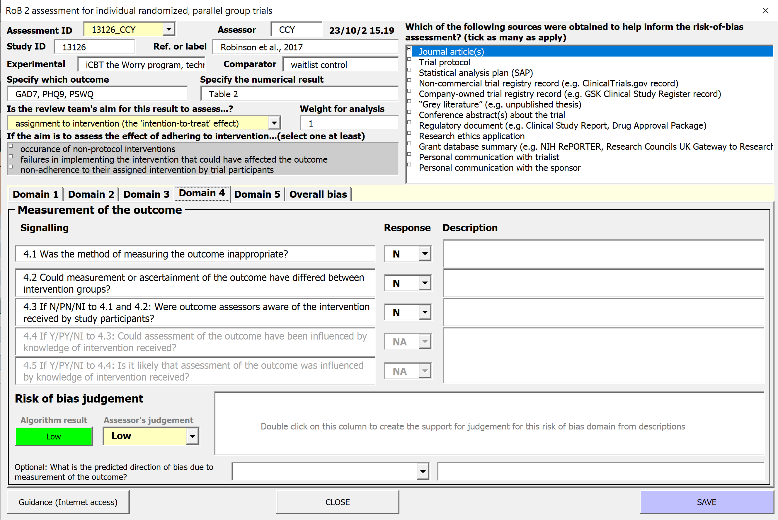

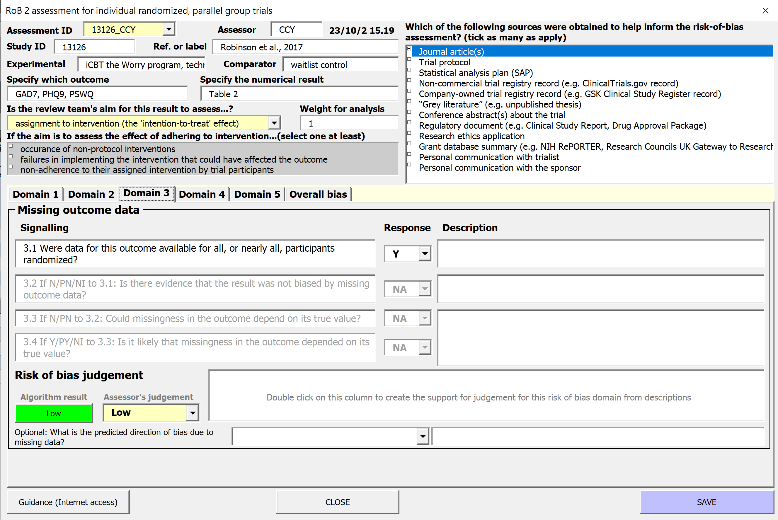

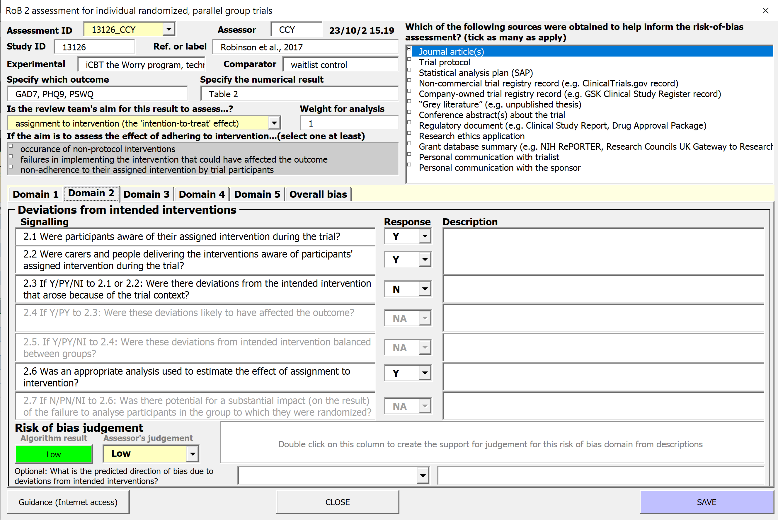

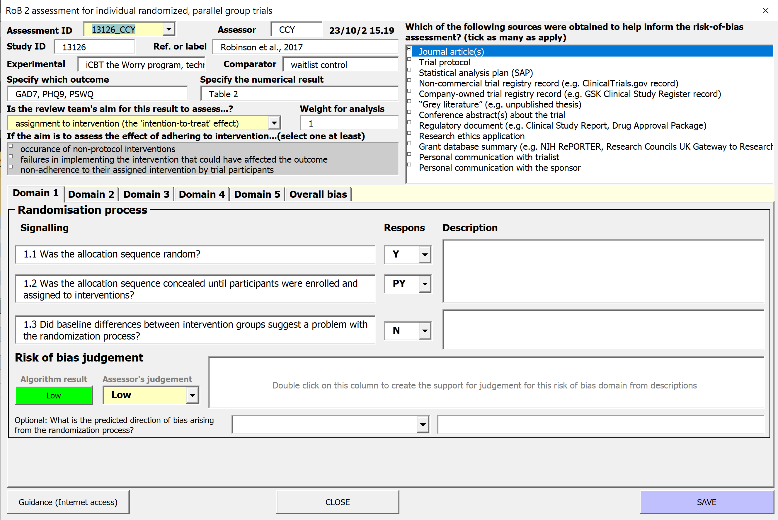

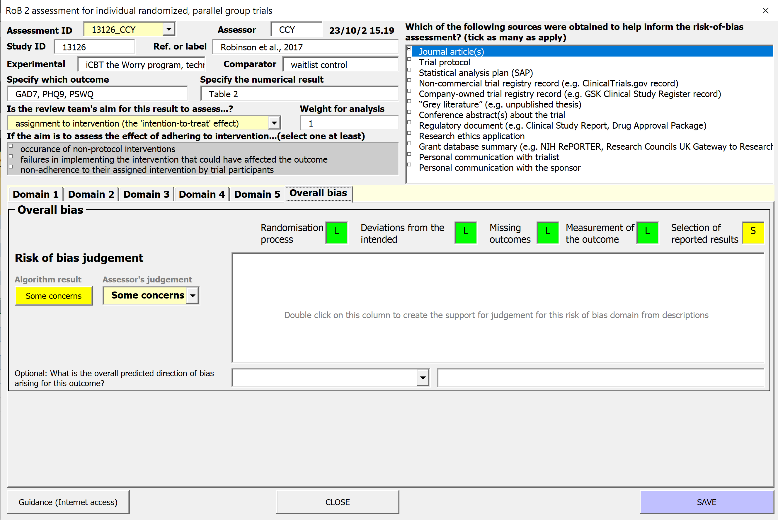


Appendix 2.10 RoB2 result of Rollman et al. (2017)


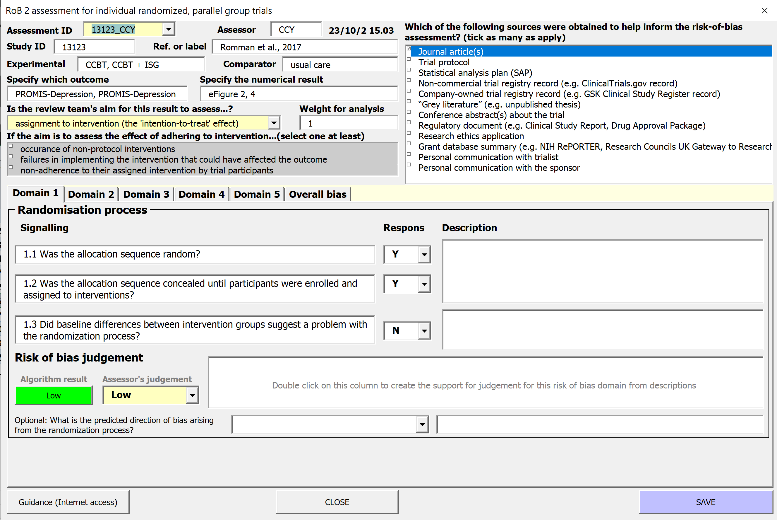

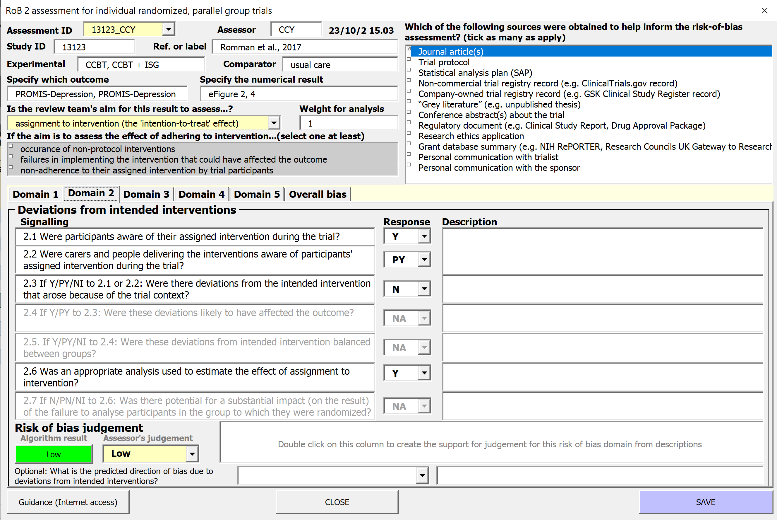

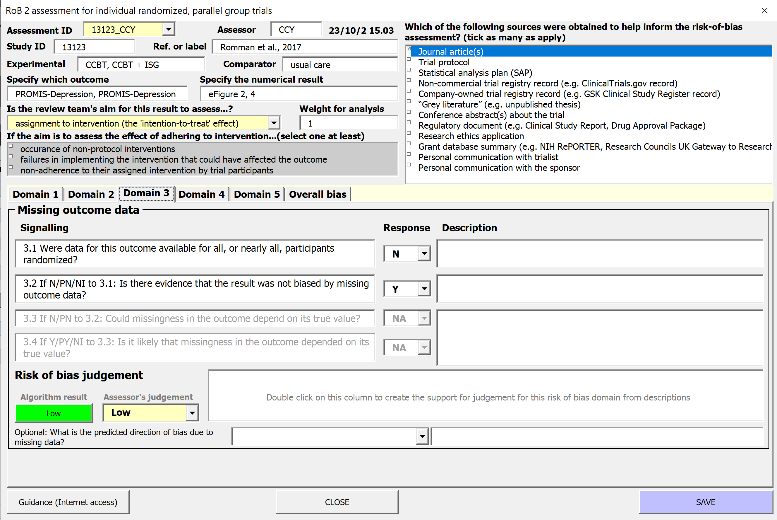

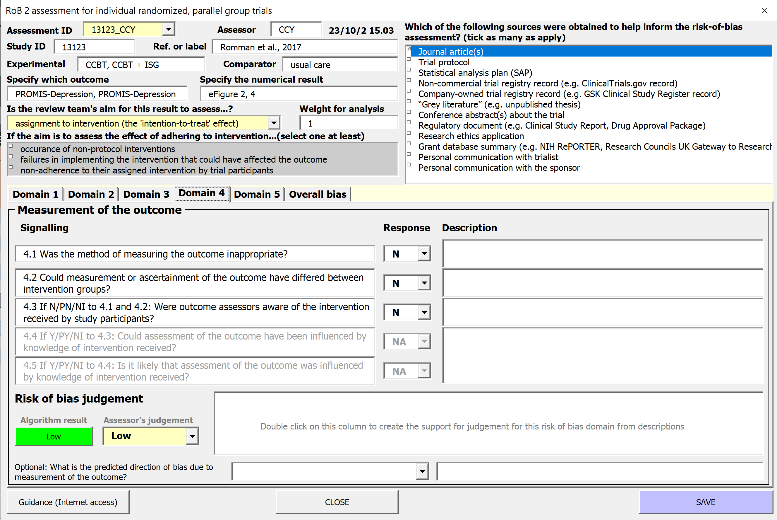

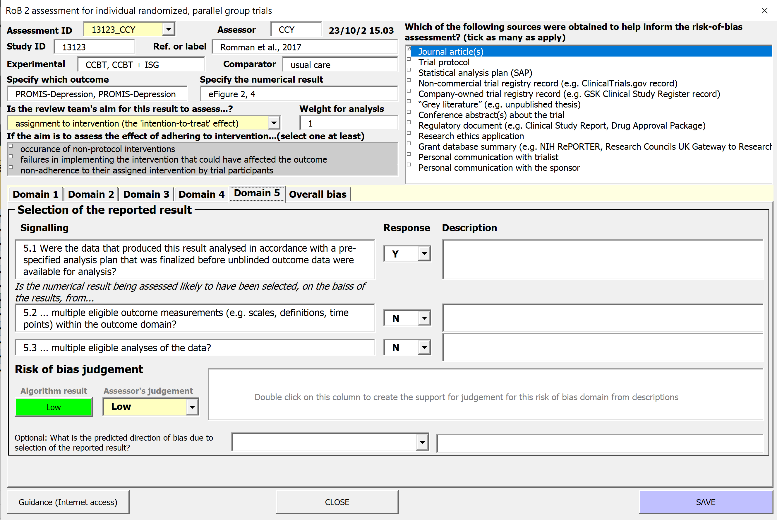

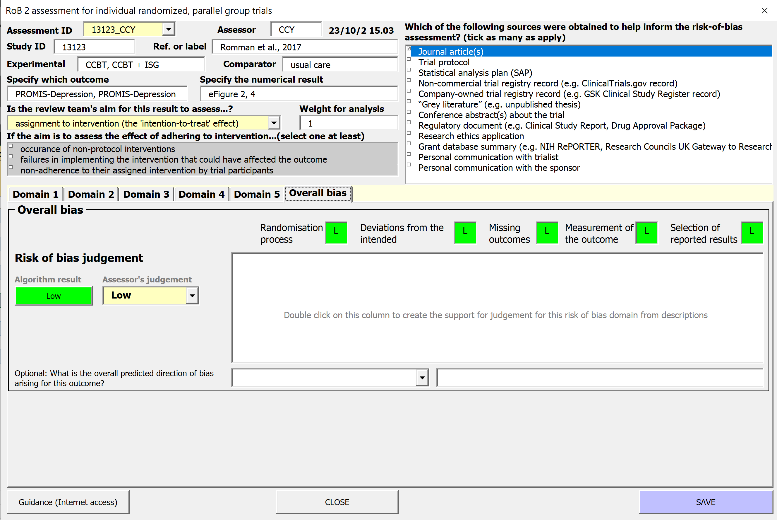


Appendix 2.11 RoB2 result of Terides et al. (2018)


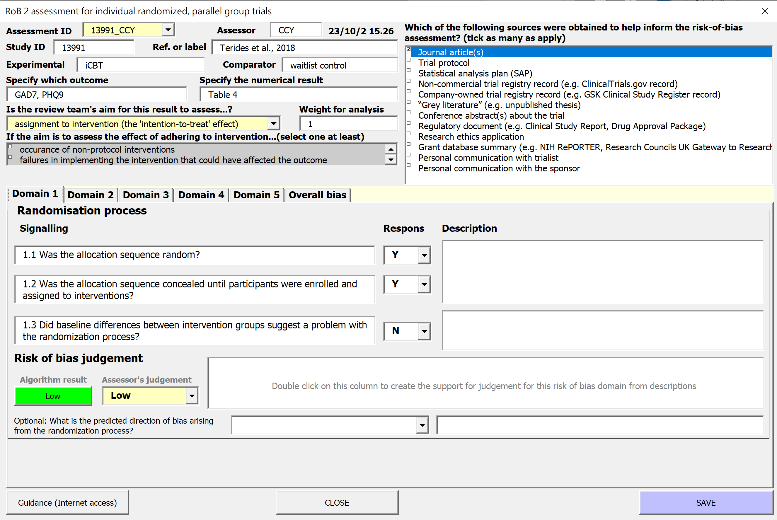

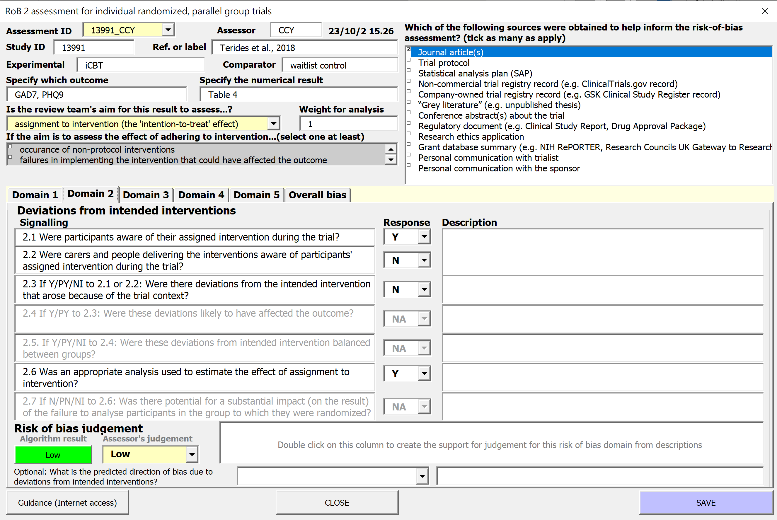

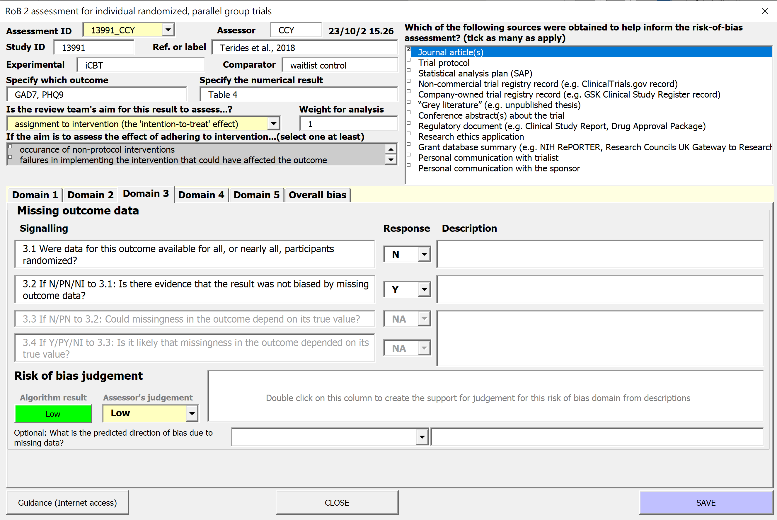

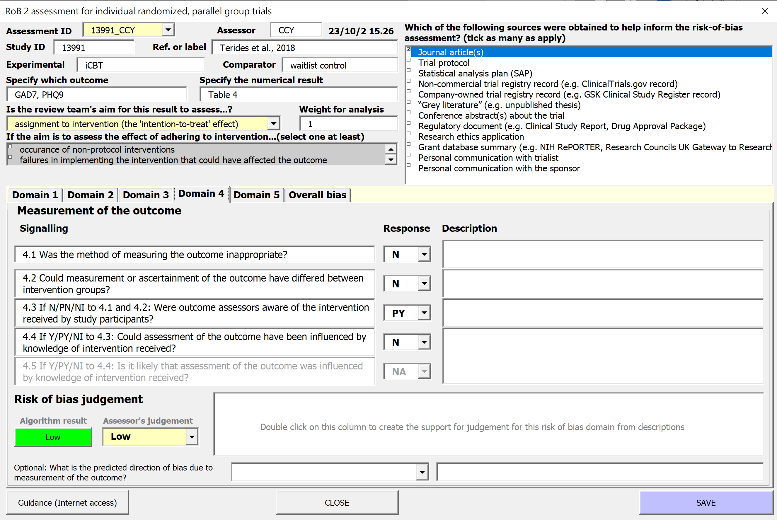

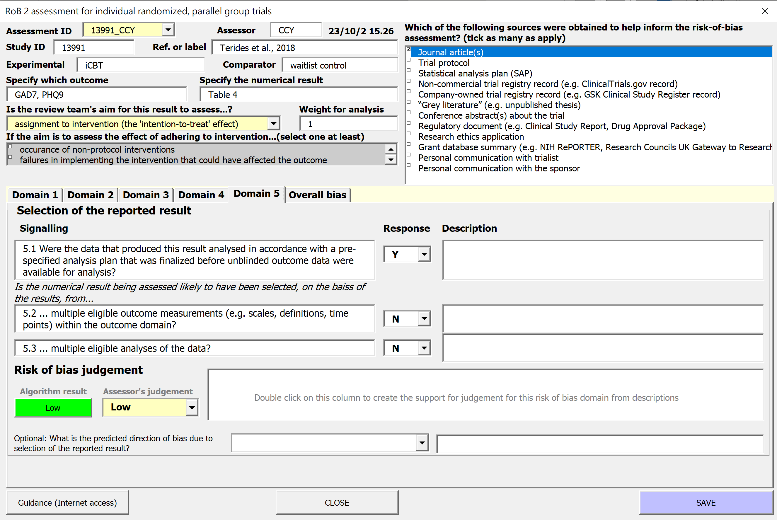

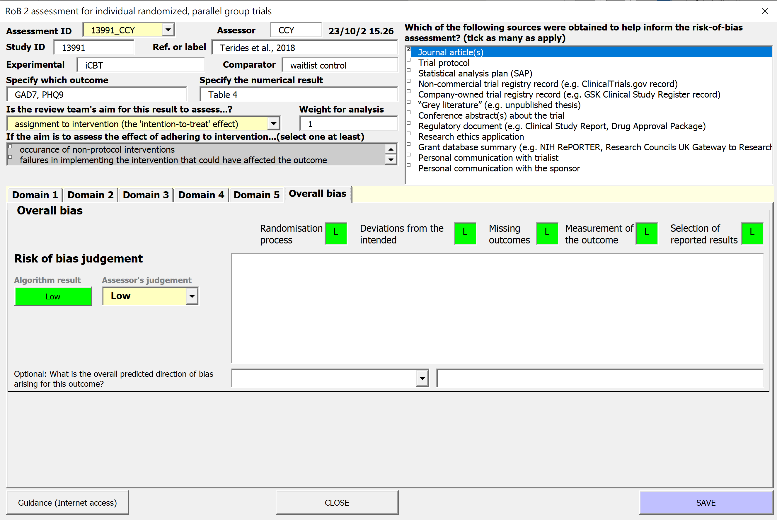


Appendix 2.12 RoB2 result of Titov et al. (2009)


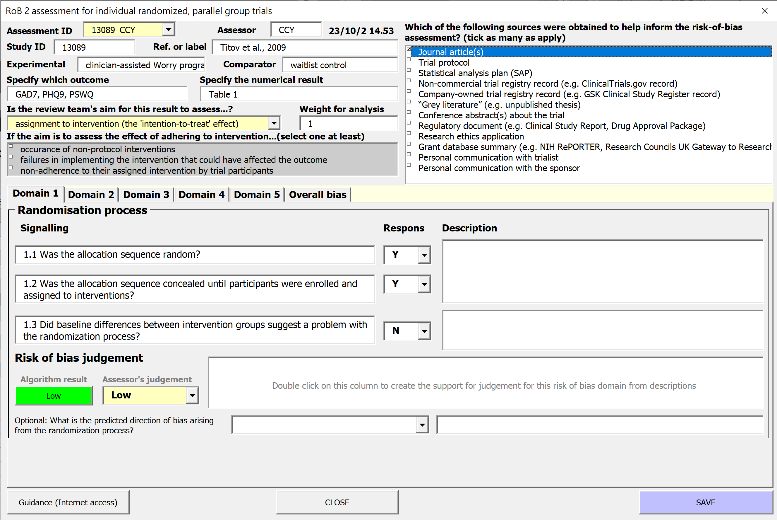

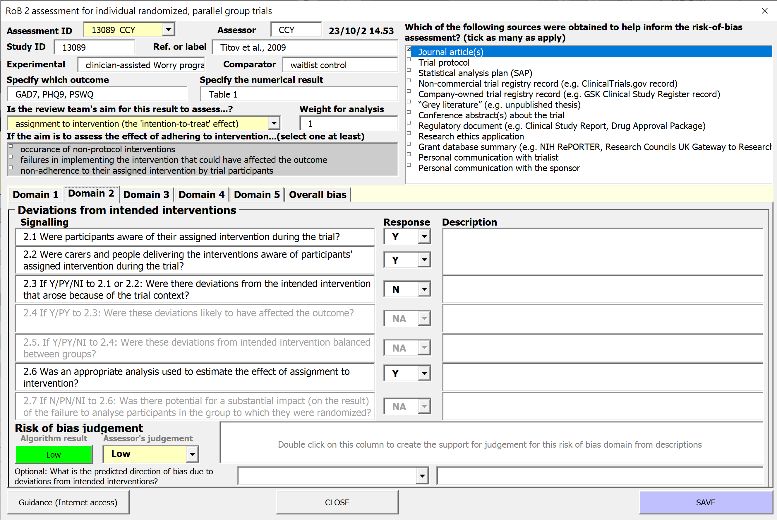

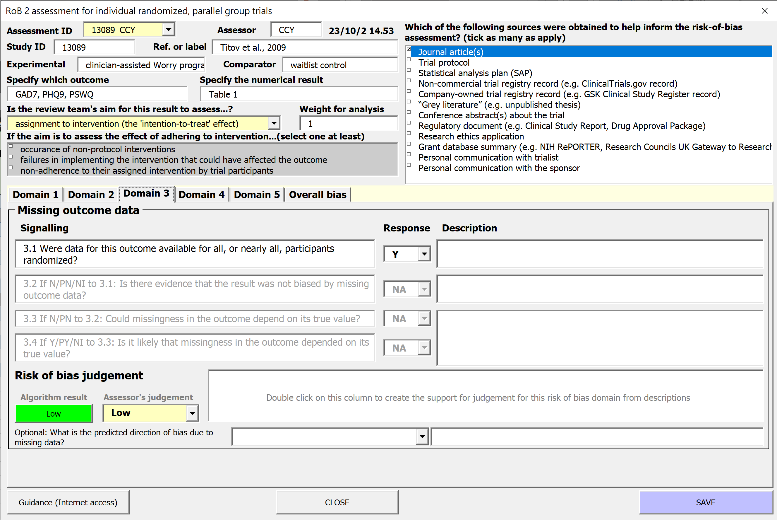

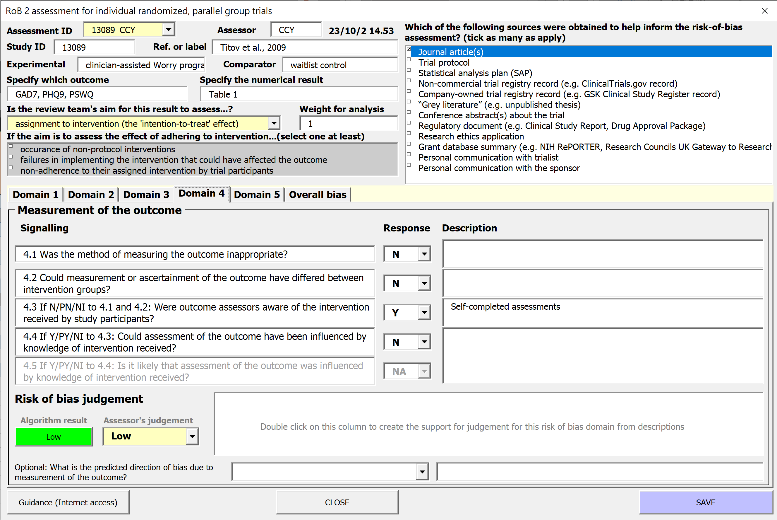

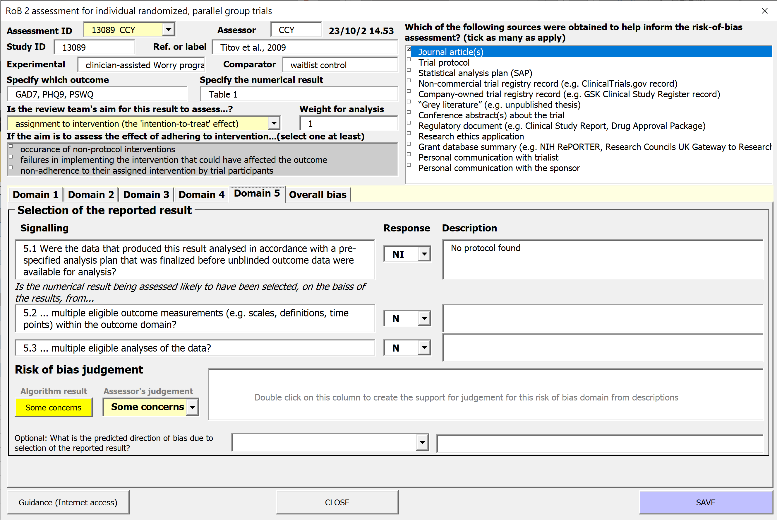

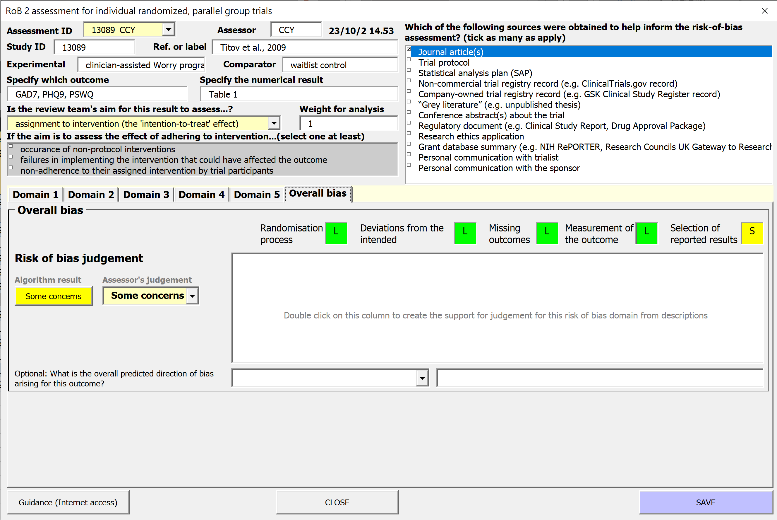


Appendix 3. Anxiety outcomes following LICBT vs. control conditions (*k* = 12)

|  |  | **Treatment** | | | | | **Control** | | | | |
| --- | --- | --- | --- | --- | --- | --- | --- | --- | --- | --- | --- |
|  |  |  | **Pre-assessment** | | **Post-assessment** | |  | **Pre-assessment** | | **Post-assessment** | |
| **Study** | **Outcome measure** | **N** | **Mean** | **SD** | **Mean** | **SD** | **N** | **Mean** | **SD** | **Mean** | **SD** |
| Al-Alawi et al. (2021) | GAD-7 | 22 | 13.59 | 4.63 | 3.77 | 3.02 | 24 | 13.50 | 3.51 | 12.29 | 4.26 |
| Andersson et al. (2012) | BAI | 27 | 24.30 | 9.10 | 15.09 | 6.99 | 27 | 25.26 | 6.03 | 59.59 | 11.47 |
| Carl et al. (2020) | GAD-7 | 128 | 15.60 | 2.80 | 9.46 | 4.67 | 128 | 15.60 | 4.07 | 11.25 | 4.70 |
| Dahlin et al. (2016) | GAD-7 | 52 | 13.83 | 3.66 | 6.90 | 3.52 | 51 | 13.51 | 2.78 | 9.13 | 4.13 |
| Jones et al. (2016) | GAD-7 | 24 | 11.78 | 4.87 | 6.50 | 4.55 | 21 | 11.99 | 7.43 | 19.20 | 8.56 |
| Newman et al. (2014) | HAM-A | 25 | 22.69 | 7.66 | 11.59 | 5.76 | 9 | 24.33 | 6.64 | 15.50 | 9.11 |
| Paxling et al. (2011) | BAI | 44 | 20.61 | 10.64 | 12.37 | 7.43 | 45 | 20.98 | 9.66 | 19.20 | 8.56 |
| Richards et al. (2016) | GAD-7 | 70 | 12.84 | 2.39 | 7.73 | 4.44 | 67 | 13.19 | 4.14 | 10.72 | 4.20 |
| Robinson et al. (2010) | GAD-7 | 97 | 12.17 | 3.76 | 5.79 | 4.10 | 48 | 12.94 | 3.10 | 12.66 | 4.70 |
| Rollman et al. (2017) | PROMIS | 114 | 69.66 | 5.04 | 59.79 | 9.53 | 41 | 68.59 | 8.59 | 15.88 | 8.87 |
| Terides et al. (2018) | GAD-7 | 43 | 14.02 | 4.41 | 7.37 | 5.69 | 46 | 12.15 | 5.15 | 7.04 | 4.81 |
| Titov et al. (2009) | GAD-7 | 24 | 14.33 | 4.50 | 6.92 | 4.40 | 21 | 13.62 | 5.08 | 10.66 | 5.46 |
| Abbreviations: BAI, Beck Anxiety Inventory; GAD-7, the 7-Item General Anxiety Disorder Scale; HAM-A, Hamilton Anxiety Rating Scale; PROMIS, Patient-Reported Outcomes Measurement Information System; SD, standard deviation | | | | | | | | | | | |

Appendix 4. Depression outcomes following LICBT vs. control conditions (*k* = 11)

|  |  | **Treatment** | | | | | **Control** | | | | |
| --- | --- | --- | --- | --- | --- | --- | --- | --- | --- | --- | --- |
|  |  |  | **Pre-assessment** | | **Post-assessment** | |  | **Pre-assessment** | | **Post-assessment** | |
| **Study** | **Outcome measure** | **N** | **Mean** | **SD** | **Mean** | **SD** | **N** | **Mean** | **SD** | **Mean** | **SD** |
| Al-Alawi et al. (2021) | PHQ-9 | 22 | 15.14 | 5.19 | 4.41 | 2.89 | 24 | 15.08 | 6.51 | 8.71 | 6.63 |
| Andersson et al. (2012) | BDI-II | 27 | 18.89 | 8.59 | 9.35 | 5.57 | 27 | 18.30 | 6.67 | 11.72 | 8.23 |
| Carl et al. (2020) | PHQ-9 | 128 | 15.40 | 5.10 | 10.80 | 6.00 | 128 | 14.70 | 5.80 | 13.15 | 6.15 |
| Dahlin et al. (2016) | PHQ-9 | 52 | 11.10 | 4.69 | 5.83 | 5.14 | 51 | 11.47 | 4.87 | 8.33 | 4.63 |
| Jones et al. (2016) | PHQ-9 | 24 | 11.65 | 7.19 | 5.59 | 5.10 | 21 | 13.20 | 6.08 | 12.08 | 6.19 |
| Paxling et al. (2011) | BDI | 44 | 17.66 | 9.81 | 10.08 | 7.75 | 45 | 16.93 | 7.91 | 16.70 | 7.60 |
| Richards et al. (2016) | BDI-II | 70 | 17.67 | 6.44 | 14.54 | 8.07 | 67 | 17.67 | 5.41 | 18.10 | 8.99 |
| Robinson et al. (2010) | PHQ-9 | 97 | 11.75 | 4.92 | 5.96 | 5.07 | 48 | 12.50 | 4.73 | 10.94 | 5.25 |
| Rollman et al. (2017) | PROMIS | 114 | 63.94 | 6.21 | 54.55 | 9.98 | 41 | 63.52 | 6.16 | 55.23 | 9.52 |
| Terides et al. (2018) | PHQ-9 | 43 | 12.12 | 4.35 | 7.34 | 5.86 | 46 | 11.89 | 5.16 | 10.34 | 4.59 |
| Titov et al. (2009) | PHQ-9 | 24 | 11.58 | 5.24 | 6.67 | 5.68 | 21 | 13.00 | 6.19 | 11.76 | 6.18 |
| Abbreviations: BDI, Beck Depression Inventory; BDI-II, Beck Depression Inventory - second edition; PHQ-9, Patient Health Questionnaire-9; PROMIS, Patient-Reported Outcomes Measurement Information System; SD, standard deviation | | | | | | | | | | | |

Appendix 5. Worry outcomes following LICBT vs. control conditions (*k* = 9)

|  |  | **Treatment** | | | | | **Control** | | | | |
| --- | --- | --- | --- | --- | --- | --- | --- | --- | --- | --- | --- |
|  |  |  | **Pre-assessment** | | **Post-assessment** | |  | **Pre-assessment** | | **Post-assessment** | |
| **Study** | **Outcome measure** | **N** | **Mean** | **SD** | **Mean** | **SD** | **N** | **Mean** | **SD** | **Mean** | **SD** |
| Andersson et al. (2012) | PSWQ | 27 | 67.89 | 6.19 | 60.78 | 9.83 | 27 | 69.74 | 5.56 | 61.88 | 7.73 |
| Carl et al. (2020) | PSWQ | 128 | 70.80 | 6.90 | 62.56 | 9.81 | 128 | 70.90 | 5.90 | 67.66 | 7.84 |
| Dahlin et al. (2016) | PSWQ | 52 | 66.88 | 7.16 | 55.29 | 10.02 | 51 | 67.45 | 6.77 | 63.35 | 8.40 |
| Jones et al. (2016) | PSWQ-A | 24 | 29.38 | 8.67 | 22.14 | 8.08 | 21 | 28.67 | 6.93 | 27.89 | 7.15 |
| Newman et al. (2014) | PSWQ | 25 | 68.81 | 7.79 | 61.58 | 10.20 | 9 | 68.89 | 8.18 | 61.00 | 10.20 |
| Paxling et al. (2011) | PSWQ | 44 | 68.74 | 5.94 | 57.82 | 13.01 | 45 | 69.32 | 6.55 | 69.39 | 7.06 |
| Richards et al. (2016) | PSWQ | 70 | 63.04 | 8.11 | 58.53 | 10.97 | 67 | 63.48 | 6.95 | 60.33 | 8.79 |
| Robinson et al. (2010) | PSWQ | 97 | 63.56 | 9.33 | 51.88 | 11.45 | 48 | 65.81 | 10.24 | 64.22 | 11.81 |
| Titov et al. (2009) | PSWQ | 24 | 66.13 | 8.25 | 56.75 | 10.78 | 21 | 66.33 | 12.70 | 66.14 | 8.70 |
| Abbreviations: PSWQ, Penn State Worry Questionnaire; PSWQ-A, Penn State Worry Questionnaire-Abbreviated; SD, standard deviation | | | | | | | | | | | |
